# Identifying wastewater chemicals in coastal aerosols

**DOI:** 10.1126/sciadv.ads9476

**Published:** 2025-05-28

**Authors:** Adam Cooper, Lucia Cancelada, Ralph Riley Torres, Kathryn Belcher, Mallory Small, Pedro Belda-Ferre, Clare Morris, Brock Mitts, Julie Dinasquet, Rob Knight, Jonathan H. Slade, Kimberly A. Prather

**Affiliations:** ^1^ Department of Chemistry and Biochemistry, University of California, San Diego, La Jolla, CA 92093, USA.; ^2^ Scripps Institution of Oceanography, University of California, San Diego, La Jolla, CA 92093, USA.; ^3^ Department of Pediatrics, University of California, San Diego, La Jolla, CA 92093, USA.; ^4^ Department of Computer Science and Engineering, University of California, San Diego, La Jolla, CA 92093, USA.; ^5^ Shu Chien-Gene Lay Department of Bioengineering, University of California, San Diego, La Jolla, CA 92093, USA.; ^6^ Halıcıoğlu Data Science Institute, University of California, San Diego, La Jolla, CA 92093, USA.; ^7^ Center for Microbiome Innovation, University of California, San Diego, La Jolla, CA 92093, USA.

## Abstract

The Tijuana River, at the US-Mexico border, discharges millions of gallons of wastewater daily—sewage, industrial waste, and runoff—into the Pacific Ocean, making it the dominant source of coastal pollution in this region. This study examines how such wastewater influences coastal aerosols by tracking spatial gradients from near the border northward. Using benzoylecgonine (a nonvolatile cocaine metabolite) as a sewage tracer, we find that wastewater compounds—including a mixture of illicit drugs, drug metabolites, and chemicals from tires and personal care products—become aerosolized and are detectable in both water and air. Spatial analyses confirm that most measured chemicals concentrate in aerosols near the Tijuana River, potentially exposing local populations to tens of nanograms per hour (e.g., octinoxate and methamphetamine) via inhalation. This airborne pathway highlights a largely overlooked source of atmospheric pollution, emphasizing the need to reassess health risks in coastal regions as global water contamination continues to escalate.

## Teaser

Wastewater-derived chemicals entering riverine and coastal waters contribute to airborne pollutants within coastal aerosols.

## INTRODUCTION

Coastal waters in the San Diego County–Tijuana border region are affected by agricultural and other pollution runoff from land, discharge of treated and untreated sewage from wastewater treatment plants, and the Tijuana River (TJR) which has become increasingly contaminated by untreated sewage and industrial waste (*1*). Although international collaboration has enabled some diversion and treatment of the TJR, precipitation in the TJR watershed can produce flows upward of billions of gallons per day that enter the Pacific Ocean in the TJR Estuary region (*2*). Increasing levels of water pollution are entering the ocean in this region, affecting densely populated coastal towns in Tijuana and San Diego County (*3*). In addition, untreated sewage released from the Punta Bandera wastewater treatment plant south of the US-Mexico border and insufficient removal of chemical pollutants in secondary wastewater plants in San Diego can result in sewage-related water and air pollution throughout the region (*4*).

Water pollution flow into the Pacific Ocean led to the County of San Diego declaring a regional public health crisis (*5*). In 2017, an estimated 3.8% of swimmers became ill from norovirus exposure in the impacted coastal region (*4*). In 2020, the US Environmental Protection Agency committed $300 million in treatment infrastructure improvements to address pollution from the TJR (*6*). To date, a comprehensive strategy for removing organic pollutants has yet to be identified (*2*, *7*, *8*). Organic contaminants of emerging concern include pharmaceuticals (*9*), illicit drugs (*7*), compounds used as active ingredients or additives in manufactured biocides (*10*), personal care products (*9*, *11*), and plastics (*12*). Many of these compounds are toxic (*11*, *13*, *14*) and pose poorly understood impacts on human health through the airborne exposure pathway (*15*–*17*).

These compounds can be introduced into the air by several pathways. In addition to ambient urban sources (*18*, *19*), organic pollutants may be emitted directly from the TJR. Volatile compounds may directly evaporate, while wind and turbulence-driven bubble formation can produce aerosols from turbulent rivers (*20*, *21*). A recent 2024 study showed that water-sourced chemical pollutants can be directly released from the heavily polluted TJR (*3*). They can also be introduced into the ocean through treated and untreated wastewater discharge, land runoff, and direct industrial release from Maquiladoras in Mexico (*22*). Once the pollutants enter the ocean, they can become transferred into the air via sea spray aerosol (SSA), as shown in both field (*15*, *23*–*25*) and laboratory studies (*26*–*28*). SSA is generated when waves break and bubbles rupture at the ocean surface, releasing chemical and biological components into the air in aerosol particles (*29*, *30*). The efficiency with which components from the ocean get transferred into the atmosphere depends on their propensity for surface enrichment (*30*, *31*) and coastal environmental conditions, including wind speed, wave height, and white cap coverage (*32*). Most SSA research has focused on the emissions of microplastics (*16*, *25*, *26*, *33*) and per- and poly-fluoroalkyl substances (PFAS) (*23*, *24*, *27*, *34*). Franklin *et al.* (*28*) identified chemicals associated with sunscreen and plastic in SSA generated from San Diego coastal seawater using breaking waves in a flume. However, limited studies exist on the broad range of chemical pollutants in ambient air near polluted coastal waters.

This study focuses on the coastal region, where, in addition to land-sourced aerosols, the river and surf zone—both heavily influenced by contaminants from the TJR—serve as a source of water-derived pollutants that become aerosolized through turbulent river regions and wave-breaking (*20*). In a previous study conducted in this same coastal region, Pendergraft *et al.* (*15*) demonstrated that a dye released in and near the mouth of the TJR was detected in coastal air at locations away from the beach. Dye detection in coastal aerosols supports the potential for coastal water pollution to transfer to locations far from the surf zone in SSA. In a subsequent study, chemicals and bacteria identified in the TJR were also detected in coastal aerosols sampled in Imperial Beach (IB) (*35*).

In this study, we report the concentrations of selected chemical pollutants in aerosols and water sampled concurrently along coastal sites in San Diego during an active sewage crisis in the TJR region before, during, and after periods of precipitation. Flow from the heavily polluted TJR increases pollutant levels in ocean water when it rains. Using a widely used sewage tracer, this study aims to better understand how and when sewage and wastewater pollution from the TJR and possibly other sources affect their airborne levels.

## RESULTS AND DISCUSSION

The primary goal of this study is to determine which chemical species measured in collected aerosols likely came from the heavily polluted TJR. We used benzoylecgonine, a widely used and robust tracer for tracking sewage pollution in water bodies (*36*), as a tracer of wastewater pollutants transferred from water into coastal aerosols. Benzoylecgonine is metabolized from cocaine (~45% of initial intake) and primarily introduced into the environment in sewage following its excretion in human urine (*37*). In comparison, around 9% of cocaine from the initial cocaine intake is excreted (*37*). Environmental degradation of cocaine in surface waters or soil can also produce benzoylecgonine, but this pathway is typically minor compared to human excretion (*38*, *39*). Cocaine’s transformation pathways in aerosols are not yet known. However, benzoylecgonine is nonvolatile, stable in the environment, and concentrates in surface waters (*40*). Therefore, detecting benzoylecgonine in aerosol samples directly indicates air masses strongly influenced by aerosols from wastewater transferred into the air.

**Fig. 1.**
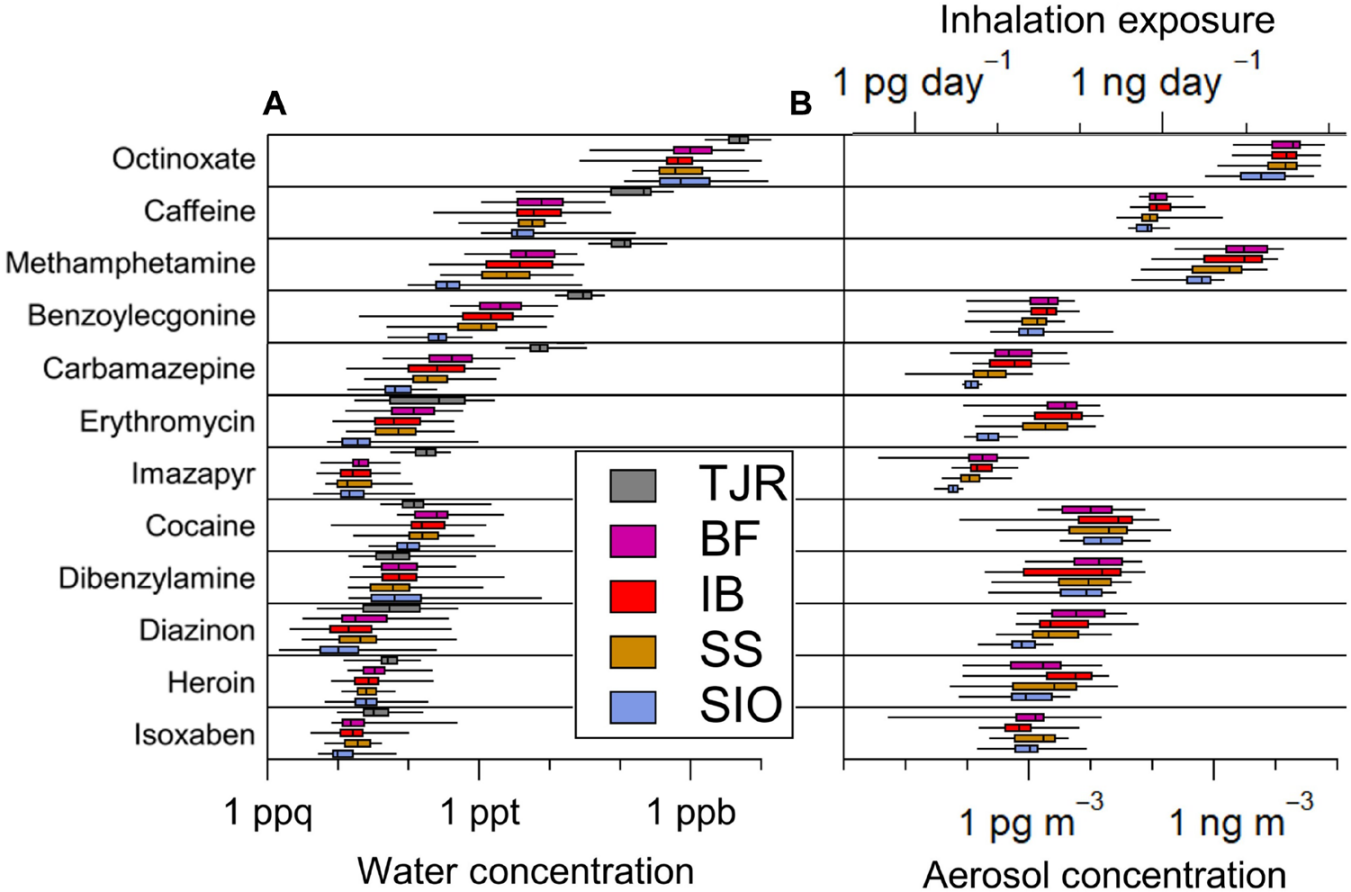
Selected wastewater chemical concentrations and spatial distributions. (**A**) River and ocean water. (**B**) Aerosol. Box plots are positioned so the vertical line indicates the median, the boxes indicate the 25th and 75th percentiles, and the whiskers represent the 5th and 95th percentiles. Compounds are organized from top to bottom based on their highest-to-lowest median concentrations in the water samples from the TJR site. Estimated inhalation exposures of aerosols on a daily basis are shown in the top horizontal axis of (B). The sampling sites are TJR, BF, IB, SS, and SIO (SIO pier). ppt, parts per trillion; ppb, parts per billion; ppq, parts per quadrillion.

This study focuses on 12 chemicals for which standards were available that represent different common categories expected in sewage, wastewater, and runoff, including personal care products, tires, illicit drugs, pharmaceuticals, metabolites, and biocides. These were quantified in aerosol samples collected over 23 hours and water samples collected daily along multiple coastal sites in San Diego and the TJR. Details of the collection, extraction, quantification, uncertainties, and data analysis procedures are provided in the Methods. Table S1 lists all selected chemicals and their median concentrations and detection frequencies in collected water samples. Median aerosol concentrations with detection frequencies are listed in table S2. For more in-depth discussion of the specific chemical constituents, including analytical challenges with the quantification of octinoxate, and concentration comparisons with other studies, the reader is referred to the texts S1 and S2. The selected chemicals’ relative water and aerosol concentrations across each measurement site are displayed graphically as box plots and plotted on a log scale in Fig. 1. Figure S1 shows the concentrations of the pollutants on the different concentration scales to better visualize differences between sampling sites. Figure S2 shows the covariation in aerosol and water concentrations across all measurement sites for which there were concurrent aerosol and water measurements. Figure S3 plots all concurrently measured aerosol and water concentrations on the same scale.

These 12 compounds exhibited concentration gradients between measurement sites in water and aerosol samples, showing the highest levels for most in water at the TJR measurement site and aerosols nearest to the river at the Borderfield State Park (BF) and IB coastal sites. The lowest water and aerosol concentrations were typically measured at a more distant coastal site at the Scripps Institution of Oceanography (SIO) (Fig. 1A). Across the entire study period, 10 of 12 of these pollutants in water showed significantly (*P* < 0.01 using the Mann-Whitney *U* test) higher concentrations in the TJR than the northernmost coastal site at SIO. The exceptions, cocaine, and the rubber additive dibenzylamine showed little variation across locations and likely have area sources from the use of cocaine and tire wear particles. Further, all 12 compounds (10 with *P* < 0.01) exhibited higher median water concentrations in either BF or IB water than in SIO water. The exceptions, octinoxate and dibenzylamine, likely have area sources from the use of sunscreen at SIO and tire wear particles. Median pollutant aerosol concentrations were generally highest at the coastal measurement sites nearest the TJR, with all 12 compounds (*P* < 0.01) exhibiting higher median concentrations in aerosols in either BF or IB air than in SIO air (Fig. 1B). This demonstrates increased airborne exposure to all measured compounds in Southern San Diego compared to SIO. See tables S3 and S4 for all *P* values.

Figures S4 and S5 refer to 10-m FLEXPART air back trajectories related to each measurement day. Table S5 reports these trajectories and precipitation conditions. Figure S6 categorizes each pollutant measured in aerosol samples according to their primary air mass origin determined from back trajectories, local wind direction data, and precipitation conditions. This analysis, however, was confounded by predominantly shifting winds, differences in atmospheric stability between day and night, and precipitation, which can lead to different levels of pollution and a mixture of offshore and onshore winds over the 23-hour collection period. Seventeen of the 23 sampling days exhibited mixed onshore and offshore winds (figs. S4 and S5), and all 3 predominately onshore sampling days at the southern locations occurred during active rainfall, which caused wet scavenging of airborne pollutants. Tables S6 and S7 list the significance levels in the differences in the concentrations of the measured chemicals between atmospheric conditions.

Two compounds, dibenzylamine and methamphetamine, had significantly higher (*P* < 0.01) concentrations on nine mixed precipitation days compared to three onshore precipitation days at the southern sites. This result is further confounded by differences in source strength, rain intensity, duration, and timing, which could not be resolved in 23-hour samples. None significantly differed between mixed and onshore precipitation days or mixed and onshore dry days at SIO. Only heroin significantly differed between two offshore dry and seven mixed dry days at the southern sites. Cocaine, benzoylecgonine, octinoxate, carbamazepine, dibenzylamine, and methamphetamine concentrations were significantly lower during three onshore days with precipitation compared to two offshore days with no precipitation. However, because of the confounding effects of wet deposition for onshore days, it is unclear from air mass origin alone whether these chemicals originated from an inland source. For these reasons, this work could not, nor does it attempt to draw conclusions for the impacts of a specific air mass origin.

These results indicate that most of the selected pollutants show higher concentrations in water and aerosols near the TJR in the BF and IB regions, dispersing and diluting to sites further to the north. However, because of the regional impact of air, as shown in a recent study tracking H_2_S emissions from the TJR (*3*), together with additional water pollution sources along the coast, the concentration gradients are relatively weak for some chemical species. To better isolate when pollution in the TJR is airborne, and to rule out multiple sources of the pollution into the air and the effects of different air masses, we now focus on tracing when wastewater was airborne by tracking and correlating these chemicals with benzoylecgonine, a marker specific to sewage water. As expected, benzoylecgonine’s concentrations were significantly (*P* < 0.0001) higher in TJR water compared to ocean water near the river mouth, higher in IB ocean water compared to ocean water at SIO (*P* < 0.0001), and higher in IB and BF aerosols compared to SIO (*P* < 0.001). This is consistent with the idea that benzoylecgonine, a wastewater tracer, has its major waterborne source from the TJR and that it becomes aerosolized predominantly from a wastewater source in the IB and BF regions.

**Fig. 2.**
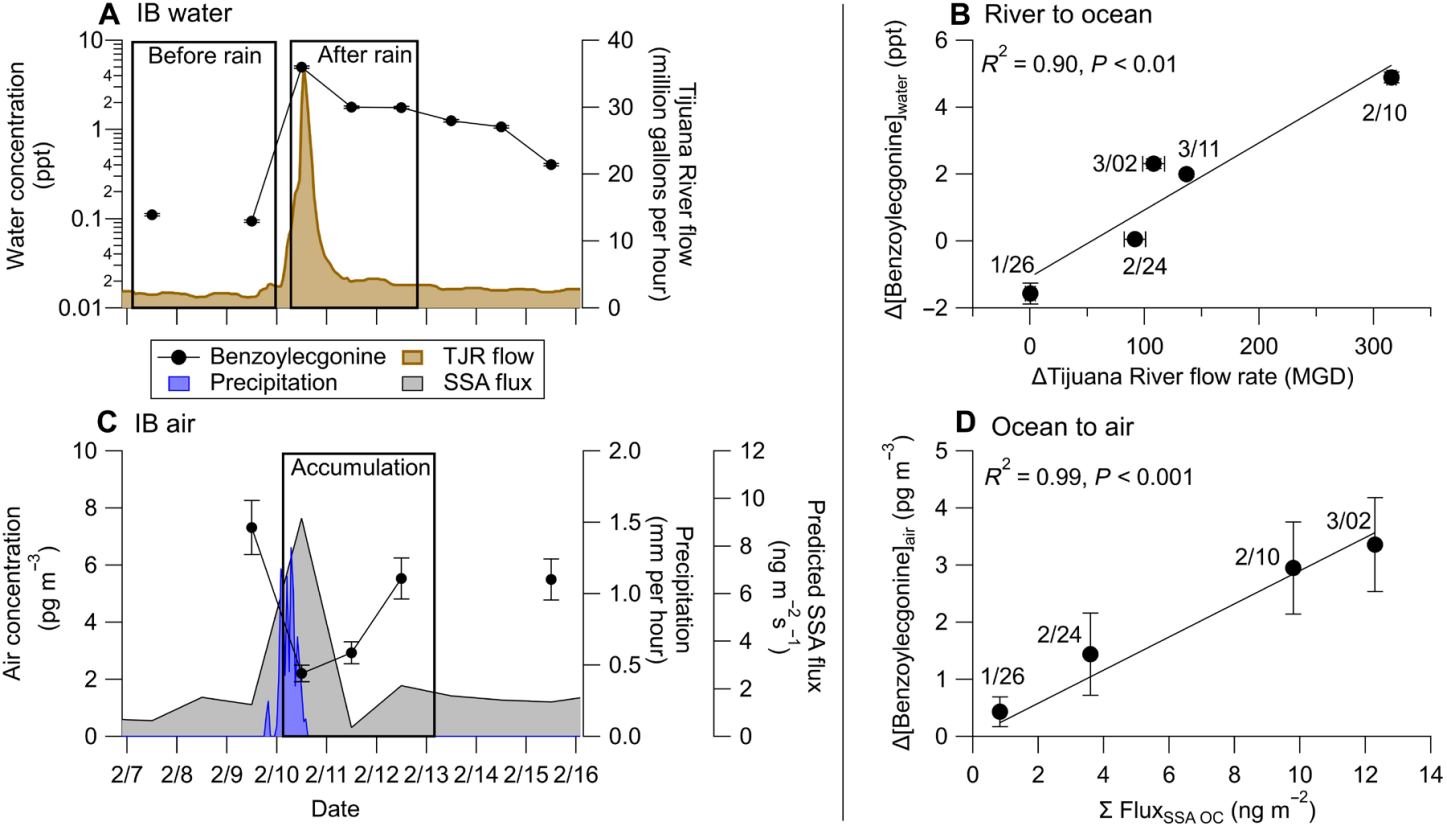
Temporal profiles of benzoylecgonine concentrations in water and aerosols at IB and their links to TJR flow and sea spray generation. (**A**) Example temporal profile of benzoylecgonine concentrations in IB water and TJR flow rates surrounding the precipitation event on 10 February. Boxes indicate the timeframe before and after the precipitation event used to calculate Δ[benzoylecgonine]_IB_ in water and the ΔTJR flow rate shown in (B), representing a dry period followed by a wet period. (**B**) The correlation between the increase in TJR flow and benzoylecgonine in IB water across all precipitation events indicates river-to-ocean transfer dependent on river flow. (**C**) Example temporal profile of benzoylecgonine concentrations in IB aerosol, precipitation rate, and predicted SSA mass flux surrounding the precipitation event on 10 February. The box indicates the accumulation period following the precipitation event used to calculate Δ[benzoylecgonine]_IB_ in aerosols and integrated predicted SSA mass flux, ∑ Flux_SSA OC_, in (D). (**D**) The correlation between the integrated modeled SSA organic carbon mass flux and the increase in benzoylecgonine in IB aerosols over the same accumulation period in (C) for all precipitation events indicates the ocean-to-air transfer of the sewage relates to SSA generation. The precipitation event surrounding 11 March was excluded from this correlation analysis because no air measurements were available to determine the concentration after precipitation ended. Error bars represent the propagated SDs in the concentrations for those days. MGD, millions of gallons per day.

To confirm this further, Fig. 2A shows the increase in benzoylecgonine concentrations in IB ocean water linked directly with precipitation-induced increases in transboundary flow in the TJR during one sampling period. Incorporating all five sampling periods (see fig. S7 for the complete time series) demonstrates a robust linear dependence between benzoylecgonine concentrations in the ocean at the IB pier and TJR transboundary flow rate (*R*^2^ = 0.90, *P* < 0.01; Fig. 2B), suggesting that the sewage-laden TJR is the major source of benzoylecgonine in IB seawater. This is also supported by the spatial gradients shown in Fig. 1 (and fig. S1), where benzoylecgonine is orders of magnitude greater in concentration in the TJR than any other water source measured. Other potential wastewater sources, although not quantified, include contributions from area sources.

It is important to note that many pollutant concentrations in the ocean increase during periods of high TJR discharge. During these periods, we detect the initial removal of benzoylecgonine in aerosols at first due to wet deposition, followed by a steady increase in concentrations after the rain event, during an “accumulation” period (Fig. 2C). Following the cessation of rain, the aerosol concentrations may increase for various reasons, including sea spray production due to high surf, resuspension of deposited particles from surfaces, and other sources such as river turbulence due to high flow conditions induced by the rain.

Our air measurements in this study are at a coastal location. Thus, we assess the connection with sea spray by comparing the increase in benzoylecgonine aerosol concentrations following periods of wet deposition to the integrated estimated SSA mass flux (equivalent to total SSA mass generated) within the accumulation period shown by the bounds in Fig. 2C. For this analysis, we used the SSA source function model developed by De Leeuw *et al.* (*32*), which parameterizes SSA generation in the surf zone using experimental data collected in San Diego (*41*). While this function overpredicts SSA flux in global models (*42*), it is appropriate for predicting increased SSA generation in the surf zone compared to the open ocean. Compared to a different model (*43*) that parameterizes SSA generation based on wind speed and sea surface temperature, the two models agree within an order of magnitude (see fig. S8 and the discussion on these calculations in text S4). In other words, the temporal trends in SSA mass flux are conserved regardless of the model, but the absolute values differ. This analysis revealed a strong correlation (*R*^2^ = 0.99, *P* < 0.01; Fig. 2D) between the increase in benzoylecgonine concentrations in aerosol and integrated SSA flux calculated over 3 days following each precipitation event, suggesting that aerosol concentrations of benzoylecgonine detected at the coastal sampling site in IB are possibly linked to SSA production.

Aerosolization along the TJR represents another potential source of airborne components from wastewater (*20*, *44*). The spatial trends identified in Fig. 1 show that sewage pollution in water and air is concentrated in southern San Diego County near the TJR outflow. While this represents initial evidence that SSA could be an important conduit for benzoylecgonine and possibly other chemicals from the polluted TJR to the atmosphere, more inland and coastal measurements are needed to determine the relative contributions from different locations.

**Fig. 3.**
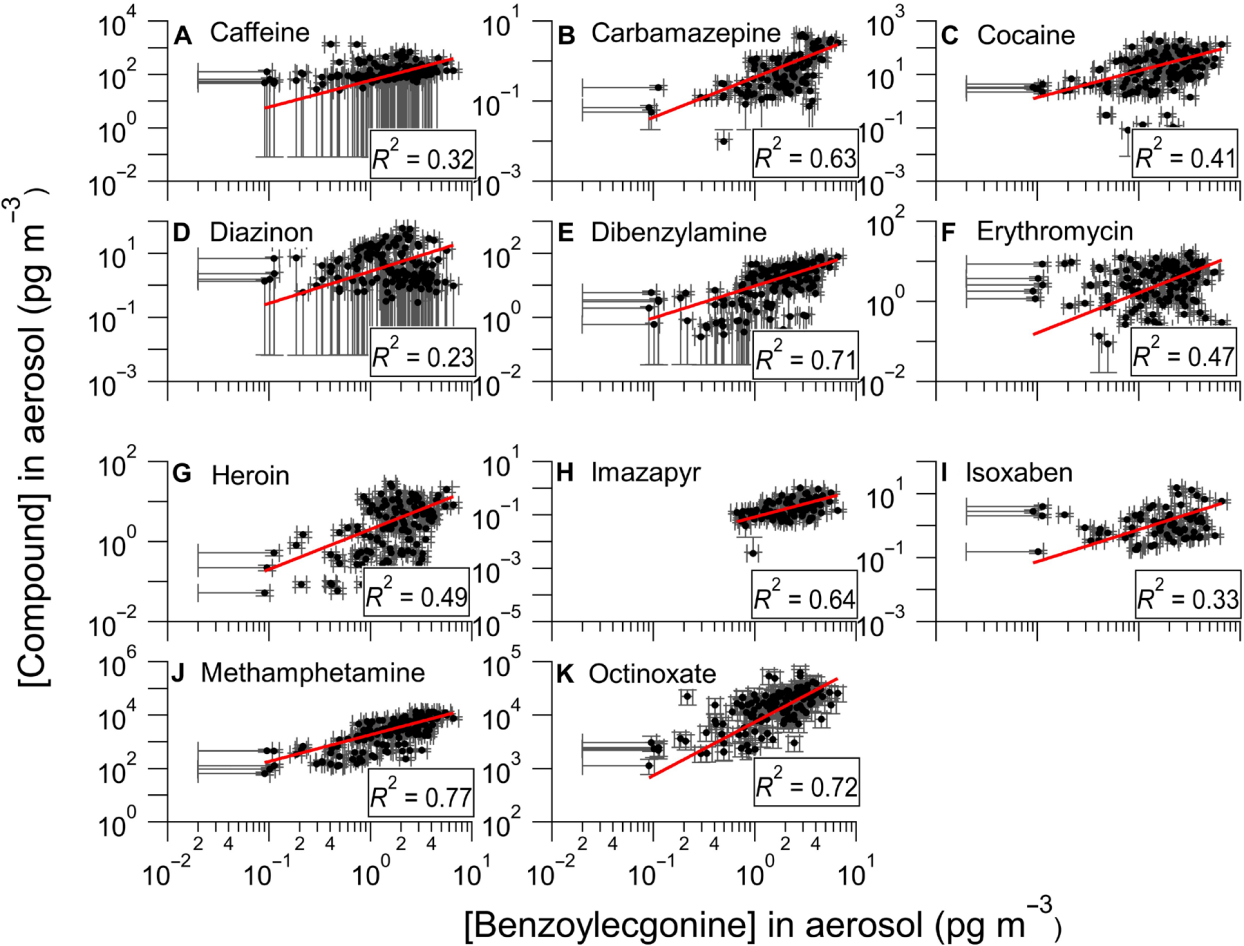
Linear regressions between the quantified pollutants and benzoylecgonine in aerosols from the BF, IB, and SS measurement sites. (**A**) caffeine, (**B**) carbamazepine, (**C**) cocaine, (**D**) diazinon, (**E**) dibenzylamine, (**F**) erythromycin, (**G**) heroin, (**H**) imazapyr, (**I**) isoxaben, (**J**) methamphetamine, and (**K**) octinoxate. Error bars reflect the relative uncertainty in the calibrated sensitivity for each compound. Horizontal error bars that extend to the left axis were below the quantification limit of benzoylecgonine and extended to its detection limit. Vertical error bars that extend to the bottom axis were below the quantification limit of the pollutant and extended to their detection limits. The intercept of the line of best fit is set to zero.

Using benzoylecgonine as an organic tracer of sewage and the understanding that, from Figs. 1 and 2, benzoylecgonine concentrations in aerosols are significantly higher in the regions (BF and IB) most affected by inputs from the TJR, positive linear correlations between the other pollutant concentrations and benzoylecgonine in the aerosol samples indicate that they were likely aerosolized from the same sewage-laden water that originated from the TJR. Figure 3 shows the linear regression analysis between the quantified pollutants in aerosols and benzoylecgonine at the southern sampling locations. Spanning two orders of magnitude in aerosol concentrations, octinoxate, methamphetamine, and dibenzylamine displayed the most robust correlation coefficients (strong correlations for *r*^2^ values > 0.7) of 0.72, 0.76, and 0.71, respectively, with *P* < 0.00001, indicating a shared sewage-laden water source with benzoylecgonine. The same analysis for the “background” samples at SIO is shown in fig. S9. However, 2 (of 20) sampling days at SIO revealed extreme outliers in benzoylecgonine concentrations in aerosols, corresponding to ~20 pg m^−3^ compared to <2 pg m^−3^ on the other 18 sampling days. This was associated with mixed onshore/offshore flow and precipitation conditions, as shown in fig. S6, although the exact reason for the anomalous concentrations is unclear.

Weaker linear correlations were observed between benzoylecgonine and some other quantified pollutants, indicating input from additional water and/or air sources. It is important to note that the relative source contributions to the river vary with different ratios from industrial waste, agricultural, sewage, and pollutant run-off, which explains the observed river and air variations, with the strongest correlations associated with species emitted in sewage. Moderately correlated pollutants, including the biocide imazapyr, pharmaceutical drugs carbamazepine and erythromycin, and other illicit drugs, including heroin and cocaine with 0.4 ≤ *r*^2^ ≤ 0.7, indicate a possible shared wastewater aerosol source with benzoylecgonine, although these correlations are too weak to link exclusively to the TJR. Both heroin and cocaine, which can be released directly into the air during consumption and when trafficking, were weakly to moderately correlated with benzoylecgonine in aerosols, with *r*^2^ values of 0.49 and 0.41, respectively. Other biocides, diazinon and isoxaben, exhibited weak correlations with benzoylecgonine, with *r*^2^ values of 0.23 and 0.33, respectively, implying additional water and/or terrestrial airborne sources besides sewage. Other possible sources include biocides in agricultural dust and evaporation and recondensation after application (*45*). Although caffeine is a common marker for human contributions to sewage (*46*), it is found in water, air, and soil. This is consistent with the observed relatively weak correlation (*r*^2^ = 0.32) between caffeine and benzoylecgonine, a tracer specific to sewage-impacted water in the air.

### Effects of chemical properties on the transfer of chemicals in wastewater to the atmosphere in coastal aerosols

**Fig. 4.**
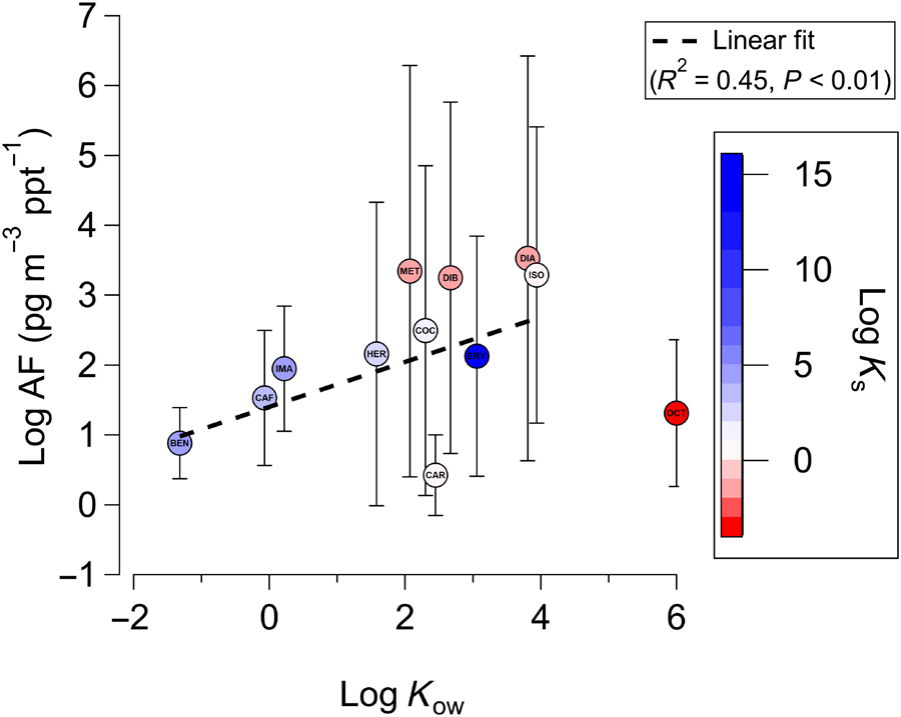
Aerosol-to-water ratios of pollutants and dependence on hydrophobicity and bubble scavenging efficiency. Log average *AF*s for each pollutant are shown as a function of their octanol-water partitioning coefficient, *K*_ow_. (*98*). Data points are colored by their scavenging coefficient, *K*_s_, defined as the ratio between the aqueous adsorption coefficient (*K*_aq_) and air adsorption coefficient (*K*_a_). BEN, benzoylecgonine; CAF, caffein; CAR, carbamazepine; COC, cocaine; DIA, diazinon; DIB, dibenzylamine; ERY, erythromycin; HER, heroin; IMA, imazapyr; ISO, isoxaben; MET, methamphetamine; OCT, octinoxate. The *AF* for OCT is not included in the linear fit due to its relatively poor scavenging by bubble entrainment. Only days at BF, IB, and SS are included in the average.

Here, we assess the efficiency of water-to-air transfer of the measured pollutants based on their chemical properties. Two factors that affect the selective transfer of organic pollutants from the ocean to the air in SSA are accumulation at the air-water interface in the surface microlayer and entrainment in air bubbles (*30*, *31*, *47*). Surface activity can be estimated by the octanol-water partitioning coefficient, *K*_ow_. While *K*_ow_ directly parameterizes hydrophobicity, it has been shown to constrain selective transfer in SSA (*30*, *48*). The selective transfer of organic compounds in SSA is also mediated by the entrainment of compounds in air bubbles that rupture at the air-sea interface to produce SSA. This process can be parameterized by the air adsorption coefficient (*K*_a_) and the aqueous adsorption coefficient (*K*_aq_) (*47*). Compounds where *K*_a_ >> *K*_aq_ will accumulate in water and not be efficiently transferred in SSA. Compounds where *K*_a_ ~ *K*_aq_ will accumulate at the bubble-water interface and be incorporated into SSA. Compounds where *K*_aq_ >> *K*_a_ will accumulate within the bubble and may transfer into the gas phase or become incorporated into SSA, depending on the compound’s volatility. The ratio of *K*_aq_:*K*_a_ is represented by the bubble scavenging coefficient, *K*_s_. To understand the efficiency of the ocean-air partitioning of these compounds, the known *K*_ow_ and calculated *K*_s_ for each quantified pollutant (parameters provided in table S8) were compared with their aerosolization factors (*AF*s) (Fig. 4), calculated as the ratio of the compound’s concentration in aerosol to its concentration in water collected on the same day (*26*). Note that this ratio of ambient concentrations serves as a proxy for aerosol enrichment from the water, and there may be other aerosol source contributions, as discussed, besides SSA. Therefore, *AF*s cannot be attributed exclusively to the compound’s enrichment in SSA. Given that we had no measurements of [Na^+^], a marker for SSA, we could not calculate enrichment to [Na^+^], as was done in other studies of SSA enrichment (*30*, *31*, *49*). *AF* is plotted directly against *K*_s_ in fig. S10.

This analysis shows a significant (*P* < 0.01) but moderate (*R*^2^ = 0.45) correlation between log *AF* and log *K*_ow_, indicating a potential link between pollutant enrichment in aerosols and the surface activity of waterborne pollutants (*47*, *48*). The uncertainties, representing 1 SD from the mean in log *AF*, reflect the variability in pollutant concentrations between measurement days and across locations. Notably, this relationship includes compounds discussed previously as likely having additional terrestrial airborne sources, including certain drugs and biocides.

Compounds with low *K*_ow_ but high *K*_s_, such as benzoylecgonine, caffeine, and imazapyr, may not accumulate at the surface but still be scavenged and emitted by bubble bursting. Compounds with high *K*_ow_ but low *K*_s_, such as octinoxate, may accumulate at the surface but not be efficiently scavenged by bubbles. Thus, they may not transfer as efficiently into SSA as other compounds, as indicated by their relatively low *AF*. For this reason, octinoxate was not incorporated into the linear regression analysis of log *AF* versus log *K*_ow_. However, its high abundance in aerosol and ocean water samples and its large *K*_ow_ demonstrate that even compounds not preferentially scavenged by bubbles may still exhibit transfer into SSA if enriched at the surface and in high abundance.

Processes beyond the scope of this study may influence the measured *AF* of octinoxate and compounds with similar properties. One potential pathway for surface-active semivolatile compounds like octinoxate is to enter aerosols through volatilization from the water surface, followed by partitioning into the particles, similar to the gas-to-aerosol transfer mechanisms observed with other polycyclic aromatic hydrocarbons (*50*). The precise transfer mechanism of octinoxate from water to air warrants further investigation. In addition, photochemical degradation of ultraviolet (UV)–absorbing compounds like octinoxate is known to occur rapidly in the aerosol phase (*51*, *52*). Degradation and volatility, combined with octinoxate’s estimated lower scavenging efficiency by bubbles, may contribute to its observed lower *AF*.

Further studies are needed to understand the factors controlling the contributions that lead to chemical lifetime in aerosols. For example, in a recent study on the photochemical degradation of the UV filter oxybenzone in marine-like aerosol particles, the estimated photochemical lifetimes of oxybenzone in aerosols were in the order of minutes compared to days in seawater (*52*). Back-of-the-envelope calculations suggest that when this representative UV filter reaches the aerosol filter inlet, its concentration may be lower by two orders of magnitude (see text S4) compared to when it was directly emitted by ocean sea spray. In this view, log *AF* for octinoxate is likely greater by a similar order of magnitude than could be discerned from the measurements and better in line with the fit in Fig. 4. Considering the heterogeneous oxidative loss rate of bisphenol-A—a common plastic residue pollutant—with an e-folding lifetime of approximately 4 days in SSA mimics at an OH-radical concentration of 1.5 × 10^6^ molecules cm^−3^ (*53*), the measured aerosol pollutant concentrations after an accumulation period following a rain event (between 3 and 4 days) could be two to three times lower than those freshly emitted in SSA. These chemical losses are difficult to quantify from field measurements but suggest that the true *AF*, determined from unreacted concentrations, for the most reactive species is greater than what can be measured in the field.

This analysis shows that waterborne pollutants originating from sewage water and runoff that enter the ocean following precipitation events can be released into the atmosphere, where they can travel long distances (*34*, *54*), persist in urban air (*55*), and affect airborne exposure to a broad range of those living near coastal communities.

#### Estimated transfer between different environmental compartments

##### River-to-ocean transfer

**Fig. 5.**
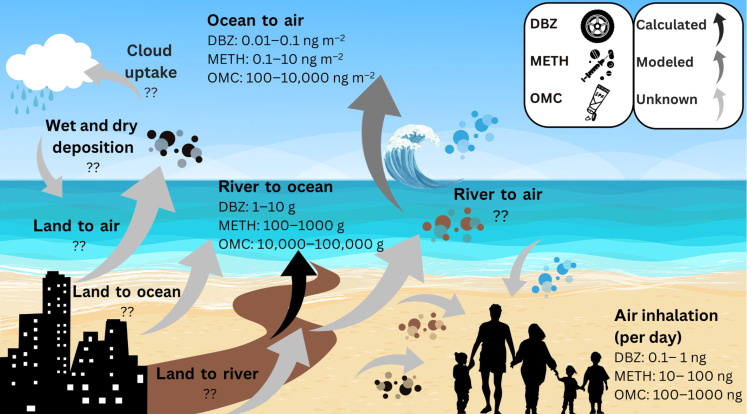
Exploratory estimated transfer of the pollutants between major environmental compartments and humans in an urban coastal environment. Reported are daily median-to-max mass transfer rates of the three most correlated pollutants (dibenzylamine, methamphetamine, and octinoxate) with benzoylecgonine, a tracer of sewage from the TJR, including river-to-ocean transfer, ocean-to-air transfer, and air inhalation. The black arrows refer to mass transfer processes that can be determined directly from the measurement data. The dark gray arrow refers to the estimated ocean-to-air flux based on IB’s measured ocean water concentrations using a predictive model relying on additional assumptions as described in the text. The light gray arrows refer to other likely intercompartmental transfer scenarios, yet the fluxes are unknown, requiring further study. DBZ, dibenzylamine; METH, methamphetamine; OMC, octyl methoxycinnamate, also known as octinoxate.

Using the specific pollutant concentrations measured in the TJR and the river flow rates, we estimated the fluxes of individual pollutants into the Pacific Ocean, as shown in Fig. 5. For this analysis, the daily flow rate for each sampling day was multiplied by each pollutant’s median and maximum concentration measured in the TJR water. The more concentrated pollutants, such as octinoxate and methamphetamine, have estimated median-to-maximum discharge rates of 10 to 100 kg day^−1^ and 0.1 to 1 kg day^−1^, respectively, while relatively lower concentration pollutants like dibenzylamine correspond to 1 to 10 g day^−1^. Since dibenzylamine is a rubber additive likely representing 1% of rubber mass (*56*), it could indicate much higher concentrations of rubber microplastics than we can calculate here. For comparison, the mass flux of octinoxate is an order of magnitude higher than wastewater influent fluxes (pretreatment) in Swiss cities (*57*). Methamphetamine median-to-maximum flux from the TJR (0.1 to 1 kg day^−1^) was slightly larger than that measured in Oslo, Norway sewage influent (142 to 295 g day^−1^) (*58*). The large riverine fluxes of these pollutants pose serious environmental concerns for surrounding riverine and marine ecosystems and the health of hundreds of thousands of residents and beachgoers frequenting areas such as IB and Playas de Tijuana. Moreover, some fish have exhibited addictive responses to methamphetamine at environmentally relevant levels [below median concentrations measured at BF, IB, and Silver Strand (SS)], altering their behavior and potentially influencing whole communities (*59*).

##### Ocean-to-air transfer of wastewater chemicals

The potential ocean-to-air transfer is modeled on the basis of the previously described SSA source function (see text S3 for details) (*32*). This analysis is done to better assess the range of concentrations of the chemicals that correlated strongest with benzoylecgonine that could be emitted from the ocean and into the atmosphere in SSA, focusing on the IB ocean water measurement data heavily affected by the polluted TJR and considering measured wind speeds in the SSA flux model. As noted before, aerosol source contributions could be other than the ocean in the measured aerosols. Therefore, the following approximates these emission fluxes for SSA based only on their measured concentrations in ocean water. The contribution of each compound to produced organic SSA mass is predicted by applying an SSA enrichment factor (i.e., [X]_SSA_/[Na^+^]_SSA_/[X]_sw_/[Na^+^]_sw_) according to that measured for PFAS (*34*). While these calculations are exploratory due to the lack of experimentally determined enrichment factors, we predict median-to-max mass fluxes transferred into SSA (shown in Fig. 5) ranging from ~10 to 100 pg m^−2^ day^−1^ for dibenzylamine to ~100 to 10,000 pg m^−2^ day^−1^ for methamphetamine and ~100,000 to 10,000,000 pg m^−2^ day^−1^ for octinoxate. For context, the median mass fluxes for dibenzylamine and methamphetamine are similar, and octinoxate’s is higher than those that Johansson *et al.* (*34*) estimated for the sum of perfluorooctanoic acid (PFOA) and perfluorooctane sulfonic acid (PFOS) compounds, which peaked at ~1 ng m^−2^ day^−1^ in similar polluted coastal regions. We have not applied this model to the other chemicals at different sites, although it is reasonable that their estimated fluxes would scale with their measured ocean concentrations shown in Fig. 1.

By assuming a well-mixed marine boundary layer height of 200 m and a steady onshore breeze of 5 m s^−1^ as done in (*25*), the onshore mass transfer of airborne contaminants can be estimated based on the airborne concentrations of each compound. We estimate a median-to-max transfer of ~1 to 10 g of dibenzylamine, ~10 to 100 g of methamphetamine, and ~100 to 1000 g of octinoxate to air over the 1 km IB coast per day under these conditions. Allen *et al.* (*25*), which investigated the sea-to-air transfer of microplastics, estimated a mass flux of 1 kg day^−1^ of airborne microplastics over a similar 1-km coastline. These calculations demonstrate that, on the most polluted days, the mass of airborne octinoxate may be comparable to the mass of microplastics entering terrestrial air masses at IB and similarly polluted areas. Over an annual and global scale, under the assumption that humans heavily affect half the world’s coastline (*60*) and that wind is blowing onshore for half the time (*25*), we estimate a potential median transfer of ~50 tons of dibenzylamine, ~8000 tons of methamphetamine, and ~40,000 tons of octinoxate per year from the marine to the terrestrial atmosphere. This exploratory extrapolation will vary by location depending on meteorological and oceanic conditions and proximity to pollutant sources. In addition, not all the measured aerosol concentrations can be attributed to SSA generated from IB ocean water heavily affected by the wastewater source from the TJR that transfers into SSA. Similarly, the estimated onshore mass transfer of the other chemicals measured in this study at different sites will scale with the masses reported here according to their relative aerosol concentrations in Fig. 1.

##### Airborne exposure

The following examines human exposure levels to the pollutants through the inhalation pathway. At a low-intensity inhalation rate of 0.7 m^3^ hour^−1^, IB inhalation levels of octinoxate and methamphetamine are approximately 1 to 10 ng hour^−1^, as indicated in Fig. 5, which is about one order of magnitude enhanced compared to SIO air. All other compounds exhibit lower exposures in the range of 0.1 to 1 pg hour^−1^, with generally higher exposures in the IB region than SIO, similar to their concentration gradients in Fig. 1. For octinoxate, this ambient concentration is comparable to levels observed in air samples collected above wastewater treatment plants (*61*) and three orders of magnitude higher than those measured in metropolitan Toronto, Canada (*18*). This finding illustrates the potential increased health exposure risk along this coastal site compared to inland urban environments for this ubiquitous toxic personal care product ingredient. It represents an understudied exposure pathway that needs to be considered in future sunscreen policy and investigation of these compounds in coastal aerosol. The estimated airborne exposure levels of methamphetamine are considerably less than a single “dose,” indicating minimal acute impact on public health. However, the health effects of chronic inhalation of octinoxate, methamphetamine, and other pollutants examined here have not been studied. This study serves as motivation to consider the health effects of airborne exposure to what has been previously considered solely waterborne pollutants.

### Broader implications for public health

This study shows that coastal aerosols near a major water pollution source may pose an inhalation risk for illicit and anthropogenic chemicals commonly only considered in water. Using benzoylecgonine as a sewage tracer, we isolate compounds in aerosols most likely that were derived from wastewater sources. After rain events in this environment, we show a correlation at a coastal site with SSA mass flux. Further studies are needed to determine other potential sources such as river spray and their spatial variations. As indicated in Fig. 5, several unknown trans-compartmental fluxes could not be estimated from the measurement data, requiring further study. Fundamental properties of aerosols, such as viscosity, which can affect the chemical lifetimes of toxic compounds, are still poorly quantified (*62*). This presents a potential health concern not only in coastal marine environments but also in regions near large freshwater bodies facing water pollution such as the Great Lakes, which can experience high winds leading to aerosolization in similar bubble rupture processes (*63*). Further, this study has implications for other areas with polluted riverine and estuary input into the ocean near urban centers, such as the Mississippi River, Yangtze River, and Ganges River Deltas, affecting even larger populations than San Diego (*64*). While aerosols may remain in the atmosphere for days to weeks, they can be removed through wet and dry deposition, affecting ecosystems and human water and food systems (*65*). This deposition can directly affect plant life and contaminate food and drinking water. To constrain the impact of pollution in this region, a comprehensive analysis of sewage component markers such as benzoylecgonine in soil, air, and water samples needs to be performed.

Local, regional, and federal air monitoring organizations should need to expand the compounds they monitor beyond the National Ambient Air Quality Standards (NAAQS) standard last reviewed in 2012 to better inform the public of their exposure to these emerging pollutants of concern and conduct long-term trend analysis to gauge whether this exposure is increasing or decreasing due to policy action (*66*). There is also a critical need for source control which is the most effective control strategy. Wastewater treatment agencies must detect a broader range of anthropogenic pollutants before the water is released into local water. Wastewater treatment plants should continue to pursue quaternary and water recycling techniques to fully remove organic pollution from wastewater before releasing it as effluent or using it as sludge (*7*, *8*, *10*, *61*, *67*, *68*). This study highlights that coastal populations are exposed to wastewater effluent released into the ocean and motivates an urgent need for improved wastewater treatment infrastructure, greener alternative treatments such as seagrass meadows (*69*) in areas with poor or no wastewater management, and consideration in any policy interventions to reduce airborne exposure to waterborne pollutants and pathogens.

As climate change accelerates the intensity of storms (*70*), more coastal locations in the US and beyond will experience severe flooding, overflowing combined stormwater-sewage systems, and increasing untreated release into oceans (*71*). Beyond the airborne transport of chemical pollutants, similar transport of pathogens from wastewater streams into rivers and the ocean will increase our susceptibility to diseases due to airborne bacteria and viruses that become aerosolized and then inhaled or ingested (*72*, *73*). An estimated 80% of all wastewater worldwide is untreated (*67*) and flows into the environment, ultimately ending in the world’s oceans (*74*). The traditional view is that this pollution is too dilute to have environmental implications. However, as this study shows, this waterborne pollution in rivers and the ocean can become aerosolized and expose billions living near the coast worldwide.

The findings in this study show that airborne exposure to wastewater chemicals needs to be factored into assessing health risks for people in and around this IB region, including at least as far north as SIO (*75*). The results have global implications as 3 billion people live within 100 km of coastlines (*76*), most of which are affected by human sewage and wastewater (*74*). This work focuses on an understudied route of airborne exposure to wastewater pollutants that affects the health of coastal populations. This study is an important step in exposing the potential for the impacts of wastewater pollutants in coastal aerosols. Further studies are underway to understand the sources more definitively (e.g., river compared to ocean spray emissions) and the factors controlling the release of chemicals from polluted rivers and oceans to quantify the impacts of these emerging water-based pollutants on human health.

## METHODS

### Sampling locations, collection, and study timeline

**Fig. 6.**
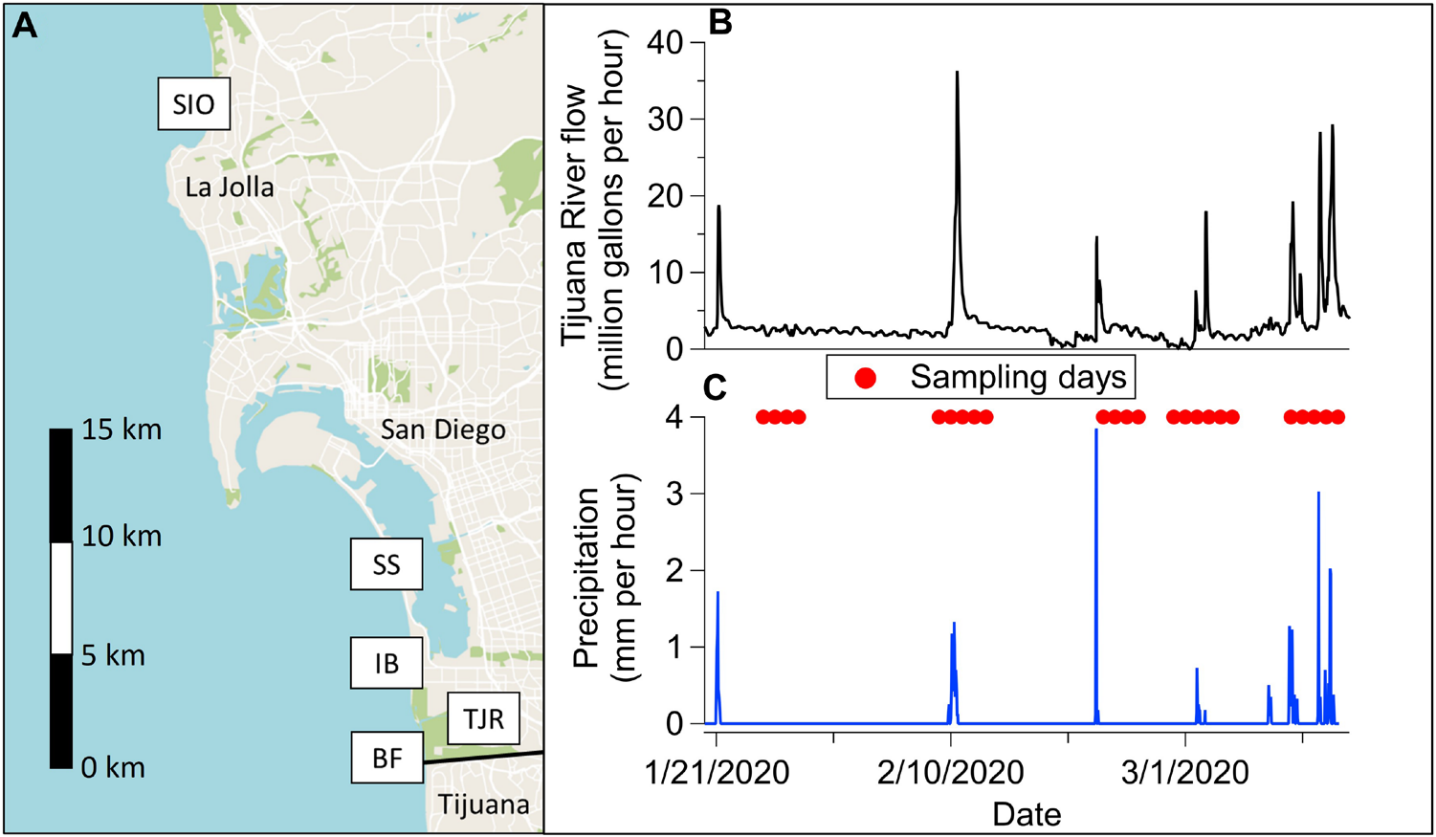
Map of sampling locations and environmental conditions. (**A**) Map of the San Diego coastal region and the border with Mexico. Field sites are labeled from south to north: BF, TJR, IB, SS, and SIO. (**B**) Hourly flow rate of the TJR measured at the international boundary (*77*). (**C**) Hourly precipitation measured at the international boundary (*77*). Sampling dates are designated as red markers.

Aerosol and water samples were collected from the surf zone on the same days at various coastal locations, starting from south to north: Border Field State Park (BF), TJR, IB, SS, and the SIO in La Jolla, CA, which served as a more distant site located approximately 35 km north of the US-Mexico border (Fig. 6A). Samples were collected surrounding periods of increased transboundary flow of the TJR (Fig. 6B) due to precipitation events (Fig. 6C).

Samples were collected between 24 January and 14 March 2020, surrounding five discrete precipitation events, where sample collection typically began a few days before rainfall and continued a few days afterward. During the sampling periods, flow from the TJR reached the ocean (*77*). Precipitation and TJR flow levels are plotted in Fig. 6. Sampling locations were chosen to observe southbound and northbound ocean and atmospheric transport of pollutants from the TJR. The first site was 2.4 km south of the TJR outflow at BF (32.5351, −117.1220). Aerosol samples were collected from a trailer 250 m from the coast from a height of 2 m above ground level (AGL). The second site was along the US portion of the TJR, 4 km inland and east of its outlet as it passes under the Hollister Street bridge (32.5514, −117.0840), whereby river samples were collected from the bridge. No aerosol samples were taken at that site. The third site was 2.6 km north of the TJR outflow at the IB Pier (32.5795, −117.1328). Aerosol samples were collected at the lifeguard tower 60 m from the ocean at a height of 3 m AGL. The fourth site was 8.7 km north of the TJR outflow at SS (32.6357, −117.1427). Aerosol samples were collected at the lifeguard tower 150 m from the coast at a height of 10 m AGL. The fifth site was 36 km north of the TJR outflow at the SIO pier (32.8661, −117.2546). Aerosol samples were collected at the pier over the surf zone at 10 m above sea level. Water samples were collected in the surf zone at all coastal sites by wading (BF and SS) or above from an available pier (IB and SIO).

Total suspended particles were collected daily on 47-mm combusted quartz-fiber filters placed in a stainless steel sample holder for approximately 23.5-hour sampling times at a nominal flow rate of ~50 liters per minute from a vacuum pump. No size selection of aerosols was performed, and gases were not scrubbed with a denuder before aerosol collection onto the filter. If semivolatile components adsorbed from the gas phase onto the quartz fiber filter, this could result in a positive sampling artifact, e.g., as seen in (*78*). One study showed that octinoxate concentrations were greater in the gas phase than in aerosols in an urban inland location (*18*). Therefore, there may be some unknown fraction collected onto the filters from the gas phase. Since there were no measurements in the gas phase in this study, no further corrections to the data have been made.

Aerosol field blanks were collected by placing aerosol filters in the sample holder and immediately replacing them. Water samples were acquired, while the filters in the aerosol sampler were changed. We used 5-gallon plastic buckets (one bucket for each site), rinsing twice with collected seawater. For each day of sample collection, water and aerosol samples were acquired roughly in order: SS [10:30 a.m. Pacific Standard Time (PST)], IB (12:00 p.m. PST), BF (3:00 p.m. PST), and TJR (5:00 p.m. PST). SIO samples were acquired roughly at 11:00 a.m. PST.

### Environmental conditions and air mass origin

Coastal ocean transport was evaluated using swell data from the Coastal Data Information Program (CDIP) IB nearshore buoy 155 (*79*). During this study, alongshore ocean currents were likely weak because swell came primarily from the west, normal to the coast (fig. S11), forcing TJR outflow to the shore with some migration to the south and north. Because of westerly swell, pollution discharge from the TJR is localized along the coastline (*1*). During this study, wave direction was primarily from the west (60%) and southwest (40%) (see fig. S11), which were predicted to drive pollution inputs to the ocean northward. However, some bidirectional and outward distribution through dilution and turbulent mixing is expected (*15*). While aerosols can be emitted from bubbles and foams formed in rivers (*20*, *21*), the aerosol composition in southern California coastal areas during onshore flows or transport over maritime regions is predominantly fresh and aged sea salt aerosol, representing more than 57% of the total particles analyzed in one study (*80*), attributed to wave-breaking and bubble-bursting processes in the ocean (*20*).

Particle origin was assessed with local winds. Local winds for the BF, IB, and SS southern sampling stations were acquired from the TJR Estuarine Research Reserve (*81*). Local winds for SIO were retrieved from the CDIP SIO nearshore station (*82*). Hourly winds during each sampling period were plotted into wind roses, as shown in figs. S4 and S5 (*83*). Because SSA may accumulate on land (*84*) and because anthropogenic pollution may accumulate over the ocean (*85*), and samples were collected for 24 hours, wind direction alone is not entirely indicative of airborne sources.

Longer-range particle sources were evaluated using the FLEXPART Lagrangian particle dispersion model (*86*). FLEXPART uses global gridded atmospheric wind data to estimate the forward or backward transport of particles or air parcels in simulated releases from a given location. Input atmospheric data came from the National Centers of Environmental Protection (NCEP) Climate Forecast System Version 2 6-hourly products, Research Data Archive, at the National Center for Atmospheric Research, Computational and Information Systems Laboratory (*87*). For each run, FLEXPART simulated the release of 1000 particles, releasing a fraction at intervals over 5 days and tracking them backward through time. Releases were simulated four times for each 23-hour sampling period, every 6 hours at 1600, 2200, 0400, and 1000 hours local time, to incorporate local, daily sea breeze patterns. One set of 96 back trajectories released particles from the IB location for the southern sampling stations, and another released particles from the SIO location. Outputs from the two release locations are similar. Particles were released just AGL, at 10 m height, and transported horizontally and vertically, according to the atmospheric winds. We limit our analysis to horizontal transport (latitude/longitude).

After reviewing the spatial coverage of the outputs, we used the 2-day data for each back trajectory, which was sufficient for visualizing and evaluating marine versus continental air mass origin. Forty-eight–hour FLEXPART back trajectories of each sampling period (figs. S4 and S5) indicate that air masses originated from terrestrial and marine locations. Our analysis attributes each sampling day to a marine, mixed, or land influence to infer potential terrestrial sources. It is important to note that the local land and sea breeze diurnal cycle, not accounted for in the long-range back trajectories, can contribute to the mixing and accumulation of background aerosol populations in the coastal atmosphere (*55*, *88*–*90*).

### Sample preparation procedures and analysis

The preparation of water samples for analysis involved the following procedure: from 5-gallon plastic buckets, 1 liter of subsamples was collected in high-density polyethylene bottles, rinsed in deionized water, and once again rinsed twice with the water sample. These 1 liter of subsamples was kept on ice throughout the sampling period from collection to laboratory preparation at ~7:00 p.m. PST. Process blanks were prepared by rinsing and transferring deionized water between the 5-gallon and 1-liter bottles. In the lab, water samples were acidified to a pH of 2 using concentrated hydrochloric acid (HCl) following the well-established protocol for dissolved organic matter (DOM) extraction described by Dittmar *et al.* (*91*)*.* Subsequently, they were stored in a 4°C refrigerator before extraction. The time between storage and extraction of the organic compounds in water samples ranged from 1 day to 1 week.

Aerosol filters were switched daily with combusted tweezers to prevent contamination, sealed in combusted aluminum foil, brought to the lab on ice, and stored in a −80°C freezer before extraction. Aerosol filter blanks (combusted quartz-fiber filters not used for aerosol collection) were handled similarly, and the time between storage and extraction of the aerosol filter samples ranged from 1 day to 1 month. For both water and aerosol samples, the amount of potential degradation during storage is unknown.

Before organic extraction, aerosol filters were thawed for 30 min and extracted with 2 ml of liquid chromatography–mass spectrometry (LC-MS)–grade methanol. Then, the eluent was brought to 40 ml with pH 2 LC-MS–grade water and agitated on a Belly Dancer orbital shaker (IBI Scientific) for 30 min. From here, organic compounds of all sample types were separated via solid-phase extraction using Priority PolLutant (PPL) resin (Bond Elut, Agilent) following the methodology described in previous works by our team (*92*, *93*). Briefly, PPL cartridges were rinsed and activated with methanol (LC-MS grade, Thermo Fisher Scientific) and conditioned with pH 2 water (LC-MS grade, Thermo Fisher Scientific). Then, water samples and aerosol filter eluent were pulled through the cartridges at a flow rate of ~10 ml min^−1^ under vacuum. After extraction, the cartridges were rinsed with pH 2 water and dried under nitrogen flow. The PPL cartridges were then stored in a −80°C freezer for 8 months before analysis due to delays from the COVID-19 pandemic. “PPL blanks” were taken by running pH 2 LC-MS–grade water through this protocol.

External standards were prepared from a mixture of 12 standard compounds for quantitative analysis and prepared at 1.37, 13.7, and 137 ng per sample, added to filters, and extracted following the same procedure as with the aerosol samples. Thus, the filter and PPL extraction efficiencies were incorporated into calibrated response factors (*92*). It is important to note, as described here in Methods, that the concentrations presented in this study were determined on the basis of external calibrations while considering the recoveries (extraction efficiencies) of the calibration standards during the filter extraction and solid-phase extraction processes, using a standard matrix without further corrections. Although the calibrations were not performed in a seawater matrix, it was assumed that the sensitivities and recoveries were consistent for both the aerosol filters and seawater samples. However, it is important to note that a previous study (*92*) reported varying recoveries for these compounds in a seawater matrix, depending on factors such as compound type, concentration, and pH, and therefore the estimates should be treated as an order of magnitude rather than quantitative.

The standard calibrations equate to 1.37 to 137 parts per trillion for water samples and ~0.023 to 2.3 ng m^−3^ for aerosol samples. Of the measured species, benzoylecgonine, caffeine, and methamphetamine were within this range for water samples; caffeine, cocaine, and methamphetamine were within this range for aerosol samples; octinoxate was primarily detected above this range for both water and aerosol samples. These calibration limits of detection are reported in table S9.

Strict quality assurance and quality control practices were followed. Aerosol field blanks were taken from each site and handled the same as filter samples. Method blanks were performed using deionized water and were handled the same way as water samples. All compounds detected (ion counts, >300) regularly (>50%) across blank measurements were discarded from further analysis.

Organic chemical analysis was performed via a nontargeted LC–tandem mass spectrometry (MS/MS) workflow detailed elsewhere (*92*, *94*) using a Thermo Vanquish Ultra-High Performance Liquid Chromatography (UHPLC) and a Thermo Orbitrap Elite Linear Ion Trap-Orbitrap (LTO) MS. Briefly, Priority PolLutant (PPL) cartridges were eluted with 2 ml of methanol, dried under vacuum, and resuspended in 100 μl of methanol/H_2_O/formic acid (80/19/1). Ten microliters of each solution was injected into the Thermo Vanquish reverse-phase UHPLC system using a Phenomenex C18 porous core-shell column. Spectra were collected using a data-dependent nontargeted method in positive mode.

Raw spectra were converted to mzXML files using ProteoWizard (*95*), and ion features were generated from extracted ion chromatograms (XIC) using MZmine2 (*96*) and linked to their MS/MS spectra, which provides a combination of relative abundance (XIC) and qualitative (MS/MS) information. Preprocessed data are available at https://massive.ucsd.edu/ProteoSAFe/dataset.jsp?task=b121936ced6d4deebd7654bb440bf56c. For peak picking in positive mode, an intensity threshold of 3000 ions for MS1 and 10 ions for MS/MS spectra were used. For the MS1 chromatogram building, a 5–parts per million (ppm) mass accuracy and a minimum peak intensity of 3000 ions were used. XIC were deconvoluted using the local minimum search algorithm with the chromatographic threshold set to 1%, a minimum relative height of 10%, a minimum absolute height of 5000, a minimum ratio of 1.5 for peak top/edge, and a minimum peak duration of 0.1 min. MS1 features were then linked to MS/MS spectra within 5-ppm mass accuracy and 0.3-min retention time windows. Isotope peaks were grouped using the isotope grouper module, and features from different samples were aligned with 10-ppm mass tolerance and 0.1-min retention time tolerance. MS1 features not accompanied by an MS/MS spectrum were not considered further. MS1 features that did not occur in at least two samples or contained a minimum of two peaks per isotope pattern, or both, were also filtered out. After filtering, gaps in the feature matrix were filled with the peak finder multithreaded algorithm with a retention time tolerance of 0.2 min, a mass tolerance of 5 ppm, and an intensity tolerance of 10%. Last, peak areas were exported in a feature table as a .csv file and corresponding consensus MS/MS spectra as a .mgf file for future analysis and processing.

The Global Natural Products Social Molecular Network (GNPS) did spectral matching and source identification (*97*). All data for this study are accessible on the GNPS site (https://gnps.ucsd.edu/ProteoSAFe/status.jsp?task=c09acc2971834bb6805121029c2003f8). Features were automatically matched to the Global Natural Products Social Molecular Network (GNPS) library spectrum, including community-provided spectra. All compounds were detected as [M + H]^+^ ions with a resolution at mass/charge ratio 200 of 140,000 (*94*). Figure S12 shows a representative mirror match for caffeine. Figure S13 shows a representative XIC plot for caffeine in a representative water, air, and standard sample. Figure S14 and table S9 show calibration curves and analytical figures of merit for the selected analytes, respectively. Each compound’s intensity and SD in solvent blanks determined the instrumental detection limits. The intensity and SD in field blanks and PPL blanks determined the method limits of detection. Calibration limits of detection were defined as three times the error in the *y* intercept of the calibration curve for each quantified pollutant.

## Supplementary Materials

### This PDF file includes:

Supplemental Text Figs. S1 to S14 Tables S1 to S9 References
