# Supplementary material for: Identifying wastewater chemicals in coastal aerosols
Source: Sci Adv. 2025 May 28;11(22):eads9476. doi: 10.1126/sciadv.ads9476 (PMC12118540; doi:10.1126/sciadv.ads9476)
Supplement: Supplementary file 1 — Supplemental Text Figs. S1 to S14 Tables S1 to S9 References [file sciadv.ads9476_sm.pdf]

Science Advances
AAAS

Supplementary Materials for

Identifying wastewater chemicals in coastal aerosols

Adam Cooper *et al.*

Corresponding author: Kimberly A. Prather, kprather@ucsd.edu; Jonathan H. Slade, jhslade@ucsd.edu

Sci. Adv. **11**, eads9476 (2025)
DOI: 10.1126/sciadv.ads9476

This PDF file includes:

Supplemental Text
Figs. S1 to S14
Tables S1 to S9
References

Supplemental text S1

Discussion of the Concentrations and Spatial Distributions of The Targeted Chemicals in Water

As shown in **fig. S1A**, the most concentrated pollutant quantified in water samples was octinoxate (octyl methoxycinnamate), an organic UV filter in sunscreens. Octinoxate and other UV filters enter coastal marine waters through wastewater effluent as well as direct rinse-off of from beach visitors.(*99*) It should be noted its quantification exhibited some challenges further explained in previous studies(*93*), its first calibration setpoint was below its LOD and not included, and had a resulting higher relative standard deviation in its sensitivity (20%) compared to other compounds (typically <3%). Octinoxate concentrations in the water samples ranged from 600 ppt at Silver Strand (SS), a site north of the Tijuana River, to almost a factor of ten higher at five ppb in the Tijuana River samples (TJR). In comparison, these concentrations are similar to levels measured off the coast of Hong Kong (89 ppt – 4 ppb)(*11*) but two orders of magnitude lower than measurements made at Trunk Bay in the U.S. Virgin Islands (1.5 ppm).(*100*) Caffeine, a well-studied chemical marker for human contributions (*46*), exhibited median concentrations ranging from 3 ppt in the ocean water at SIO to 200 ppt in river water at the TJR site. For comparison, the riverine concentrations of caffeine measured in this study are on the same order of magnitude as other measurements in rivers such as the Thames (London, UK), Hai (Tianjin, China), and the Lambro (Milan, Italy).(*101*) Ocean concentrations were typically on the lower end of other reported measurements, measured as high as 11 ppm in the Darwin Harbor of Northern Australia.(*102*) Caffeine showed a slight gradient of decreasing concentrations from the southernmost sites

moving northward to SIO, similar to other quantified pharmaceuticals.

Another water-derived compound measured was the illicit drug methamphetamine, which exhibited median concentrations of 100 ppt in river water at the TJR site of 100 ppt. In contrast, concentrations in ocean water ranged from 5 ppt at BF to 350 ppq at SIO. These concentrations were the same magnitude reported in many wastewater-influent samples and two orders higher than the Llobregat River (Barcelona, Spain).(*7*) Similar gradients were observed with other illicit drugs, including heroin and cocaine, which showed overall lower concentrations in water (<100 ppq). Cocaine's metabolite, benzoylecgonine, was also detected in the Tijuana River, showing a gradient in median concentrations with the highest concentrations in the TJR (29 ppt) followed by BF, IB, and SS (1-2 ppt) and SIO (290 ppq) suggesting the Tijuana River as a major point source.

Concentrations of the biocides imazapyr, diazinon, and isoxaben were relatively low in the water samples (<100 ppq) but exhibited strong concentration gradients between the TJR site and others, which suggests the Tijuana River as a major source. These compounds have not been previously reported in the TJR but are similar in magnitude to some that have been reported (endosulfan - 29.7 ppq and endrin - 10 ppq) and lower than others such as DDT - 2 ppt. (*103*)

Dibenzylamine, a vulcanization activator used primarily in the rubber processing industry, showed less variability in concentration (~100 ppq) between TJR water and all ocean samples. This compound is a component of rubber tires with a possible emission source from road wear,(*56*) which is a significant source of microplastics.(*104*) Notably, water concentrations in this study were much lower than previous measurements of dibenzylamine (400 ppt) in the polluted Cochin Backwater in Kerala, India.(*105*) The lack

of a gradient between TJR and ocean samples suggests that the TJR is not an important point source of this compound to the ocean.

Supplemental text S2

Discussion of the Concentrations and Spatial Distributions of The Targeted Chemicals in Aerosols

Median aerosol concentrations with detection frequencies are listed in **table S2** and displayed graphically in **figure S1B**. Median concentrations of octinoxate in aerosol ranged from ~1-10 ng m^{-3}. These ambient concentrations are comparable to levels observed in air samples collected at wastewater treatment plants(*61*) and three orders of magnitude higher than those measured in other urban locations.(*18*) Octinoxate has also been detected but not quantified in Caribbean trade winds.(*106*) To our knowledge, these are the first reported aerosol concentrations of octinoxate at a coastal urban site, underscoring the potential increased inhalation exposure risk for this toxic sunscreen chemical at coastal sites compared to inland urban environments.

Airborne concentrations of methamphetamine (~500-3000 pg m^{-3}) and caffeine (~100 pg m^{-3}) were significantly higher than those measured by Johnson et al. 2023(*107*) in urban air in Auckland, New Zealand, a city with a population similar to San Diego. They reported average methamphetamine concentrations of ~25 pg m^{-3} and caffeine concentrations of ~15 pg m^{-3}. The observed factor of 5-120 times enhancement demonstrates how the presence of untreated sewage from the Tijuana River may increase airborne exposure of these compounds.

Methamphetamine showed a strong concentration gradient at different locations, with both sites close to Imperial Beach (BF and IB) enhanced by a factor of five compared to the SIO background site. Interestingly, airborne methamphetamine concentrations were generally higher than caffeine concentrations, even though caffeine showed higher

concentrations in ocean water.

Diazinon was the biocide with the highest airborne concentration at BF of 5.9 pg m^{-3}, significantly less than airborne concentrations following immediate application (10 μg m^{-3}).(*108*) It exhibited a decreasing trend from BF to SIO, where the median concentration was an order of magnitude lower at 780 fg m^{-3}. The other biocide compounds had similar concentration gradients across all coastal sites, suggesting a local airborne source.

Median pollutant aerosol concentrations were generally highest near the TJR site with 13 of 14 compounds exhibiting higher median concentrations in IB air compared to SIO air. This gradient was most evident for pollutants associated with wastewater transported in the Tijuana River relative to more dilute ocean samples, including octinoxate, methamphetamine, benzoylecgonine, erythromycin, imazapyr, and diazinon. However, some pollutants in aerosol exhibited less of a discernable relationship to proximity to the Tijuana River, including caffeine, cocaine, dibenzylamine, and isoxaben. This may be attributed to different water and airborne sources along the coast, such as treated wastewater, runoff in other rivers, or terrestrial aerosol sources. For example, while benzoylecgonine median concentrations in water and aerosol decreased at the northernmost site further from the wastewater released in the Tijuana River, its precursor, cocaine, had no distinct spatial concentration gradients in water or aerosol. This supports the choice of benzoylecgonine being used as a primary wastewater tracer (*40, 109*), whereas cocaine can come from other non-sewage sources (*109*) in addition to wastewater release followed by aerosolization.

Supplemental text S3:

Flux calculations

We estimate the SSA flux of compounds using the updated DL00 source function from De Leeuw *et al.* 2011 (*32*), which is appropriate for estimating fluxes of SSA in the surf zone below 9 m s^{-1}, as in this study. Of note is that this parameterization was conducted at the Scripps pier, one of our sampling locations, making it particularly well suited for use in this study. The function shown in eq. 1 calculates number flux as a function of D_p (particle diameter) and U_{10} (wind speed at 10 m):

$$\frac{dF_N}{dD_p} = 4e^{0.23U_{10}}U_{10}^{3.41}D_p^{-1.5} \qquad eq\ 1$$

We modify this to mass flux assuming a spherical particle and a density of 2.2 g cm^{-3}(*110*):

$$\frac{dF_M}{dD_p} = \frac{16}{3}\pi e^{0.23U_{10}}U_{10}^{3.41}D_p^{1.5} \qquad eq\ 2$$

We separately integrate the source function for three modes of aerosol identified in Salter *et al* 2015 (D_p=0.095, 0.6 and 1.5 um)(*43*) to calculate the summed volume flux of each mode:

$$Flux_{SSA,mode\ 1} = \int_{.045}^{0.2}\frac{16}{3}\pi e^{0.23U_{10}}U_{10}^{3.41}D_p^{1.5} = 0.12e^{.23U_{10}}U_{10}^{3.41} \qquad eq\ 3$$

$$Flux_{SSA,mode\ 2} = \int_{0.35}^{1.03}\frac{16}{3}\pi e^{0.23U_{10}}U_{10}^{3.41}D_p^{1.5} = 6.73e^{.23U_{10}}U_{10}^{3.41} \qquad eq\ 4$$

$$Flux_{SSA,mode\ 3} = \int_{0.94}^{2.4} \frac{16}{3} \pi e^{0.23U_{10}} U_{10}^{3.41} D_p^{1.5} = 54 e^{0.23U_{10}} U_{10}^{3.41} \qquad eq\ 5$$

We then calculate the total Na^+ produced in each mode by applying the mass fraction of Na^+ from Bertram *et al* 2018(*29*):

$$Flux_{Na^+} = 0.055 * Flux_{SSA,mode\ 1} + 0.055 * Flux_{SSA,mode\ 1} + 0.26 * Flux_{SSA,mode\ 1} \qquad eq\ 6$$

As well as the total OC produced by applying the following:

$$Flux_{OC} = 0.82 * Flux_{SSA,mode\ 1} + 0.82 * Flux_{SSA,mode\ 1} + 0.08 * Flux_{SSA,mode\ 1} \qquad eq\ 7$$

For comparison, we also calculated SSA flux using the following parameterization from Salter *et al* 2015(*43*), where F_{U10} is the volume flux in $m^3\ m^{-2}\ s^{-1}$, N_i is the number flux of the i nm mode in # $m^{-2}\ s^{-1}$, and T is the sea surface temperature in °C. A, B, C, and D are experimentally determined fitting parameters.

$$F_{U_{10}} = 2 * 10^{-8} * (U_{10})^{3.41} \qquad eq\ 8$$

$$N_i = F_{U_{10}} * (A_i * T^3 + B_i * T^2 + C_i * T + D_i) \qquad eq\ 9$$

The Na^+ and OC fluxes were calculated using eqs. 6 and 7. The estimates using the updated DL00 parameterization generally agreed within an order of magnitude, so we decided to use the updated DL00 estimates for the remainder of the analysis.

To estimate the increase in water concentrations due to wet deposition, we use the following equation where $\Delta[X]_{air}$ is the decrease in airborne concentrations, h_{air} is the height of the marine boundary layer (estimated at 200 m here,) h_{water} is the height of the surfzone (estimated at 10 m), and ρ_{SW} is the density of seawater (~1,025,000 g m^{-3}):

$$\Delta[X]_{water} = -\Delta[X]_{air} * \frac{h_{air}}{h_{water}} * \rho_{SW} \qquad eq\ 9$$

To estimate the ocean-to-air transfer of each compound, we use the following equation from Johansson *et al* 2019(*34*) where EF is the enrichment factor of each compound, $[X]_{sw}$ is the concentration in seawater, and $[X]_{particle}$ is the concentration in SSA:

$$[X]_{particle} = EF_X * \frac{[X]_{sw}}{[Na^+]_{sw}} * [Na^+]_{particle} \qquad eq\ 11$$

We calculate the mass flux release from the TJR using eq. 1, where $Flux_{TJR}$ is the mass flux in kg day^{-1}, $[X]_{i,TJR}$ is the concentration of compound X on day i in the TJR in ppt, and $Flow_{i,TJR}$ is the flow in units of million gallons day^{-1}:

$$Flux_{TJR} = 3.8 * 10^{-3} * [X]_{i,TJR} * Flow_{i,Tijuana\ River} \qquad eq\ 12$$

We calculate the $Flux_{onshore}$ in g day^{-1} for compound X on day i using equation 8 from Allen *et al* 2020 (*25*), where $[X]_{i,IBA}$ is the concentration of compound X on day i in IB air in pg m^{-3}, U_{wind} is the average wind speed(*111*), and h_{MBL} is the height of the marine boundary layer (200 m.)(*21*)

$$Flux_{onshore} = [X]_{i,IBA} * U_{wind} * h_{MBL} \qquad eq\ 13$$

We calculate the $CTE_{i,X}$, central tendency exposure, for compound X on day i, using eq. 7, where $[X]_{i,IBA}$ is the concentration of compound X on day i in IB air in pg m^{-3}, and IR is inhalation rate (parameterized as 0.7 m^3 hr^{-1} in the EPA Exposure Factors Handbook)(*112*):

$$CTE_{i,x} = [X]_{i,IBA} * IR \qquad eq\ 14$$

Supplemental text S4:

Photochemical Lifetime Calculation

The corrected Log AF for octinoxate was calculated from the photochemical lifetimes of oxybenzone, another UV filter, in marine-like particles, as measured by Cooper et al., 2024.(*52*) Here, the effective photolysis rate, j_{eff} is 2-11×10^{-3} s^{-1}. The residence time to reach the aerosol sampling inlet is estimated to be 10 minutes. We can use the following calculation to determine the scaling factor, which scales the measured UV filter concentration in aerosol to its concentration before photochemical loss. The decay in UV filter concentration, [UV], takes the following form:

$$[UV]_t = [UV]_0 e^{-j_{eff}t}$$

Re-arranging leaves

$$[UV]_0 = [UV]_t \frac{1}{e^{-j_{eff}t}}$$

Substituting for j_{eff} and *t* yields a scaling factor of 735, assuming j_{eff} =11×10^{-3} s^{-1}.

a scaling factor of 735, assuming j_{eff} =11×10^{-3} s^{-1}.

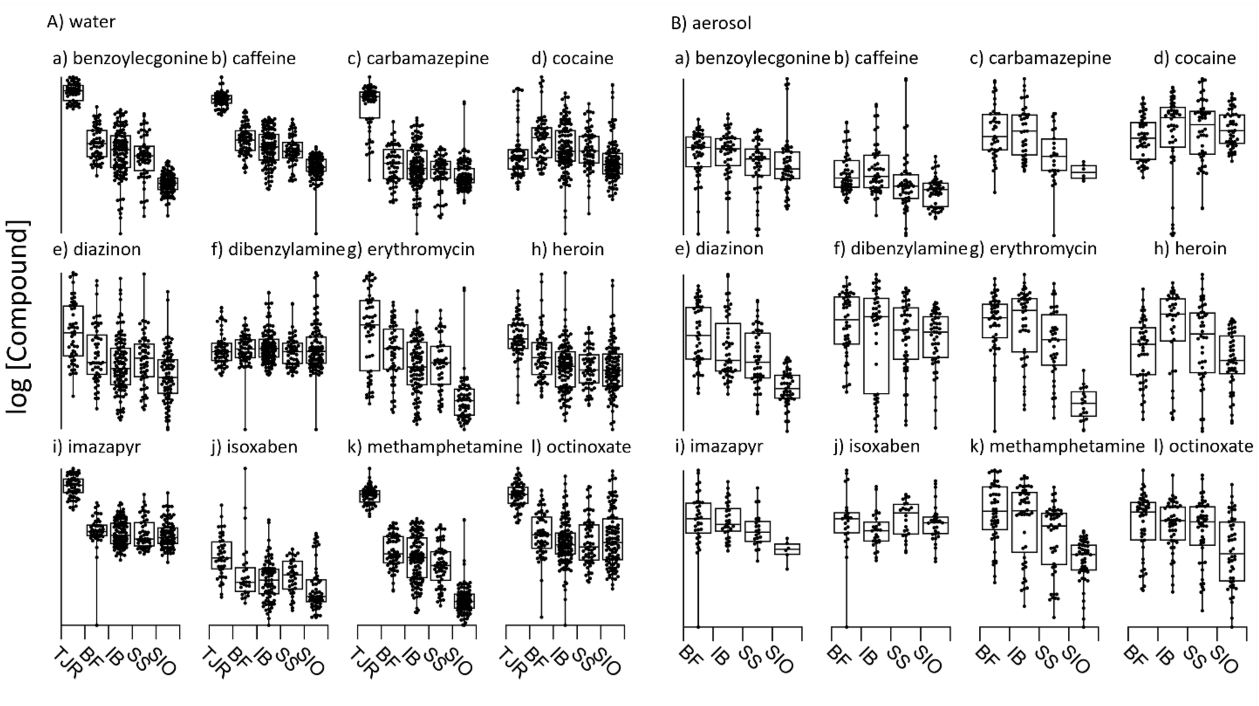

Figure S1. Relative concentrations between measurement sites. A) river and ocean water. B) aerosol. Box plots show the median as a horizontal line, the boxes indicate the 25th and 75th percentiles, and the whiskers represent the 5th and 95th percentiles. Absolute concentrations are plotted in Fig. S1. The measurement sites include TJR (Tijuana River) for river water only (i.e., no aerosols), BF (Borderfield State Park), IB (Imperial Beach), SS (Silver Strand State Park), and SIO (Scripps Institution of Oceanography pier) for ocean water and aerosol samples. These measurement sites are arranged left-to-right in a south-to-north gradient.

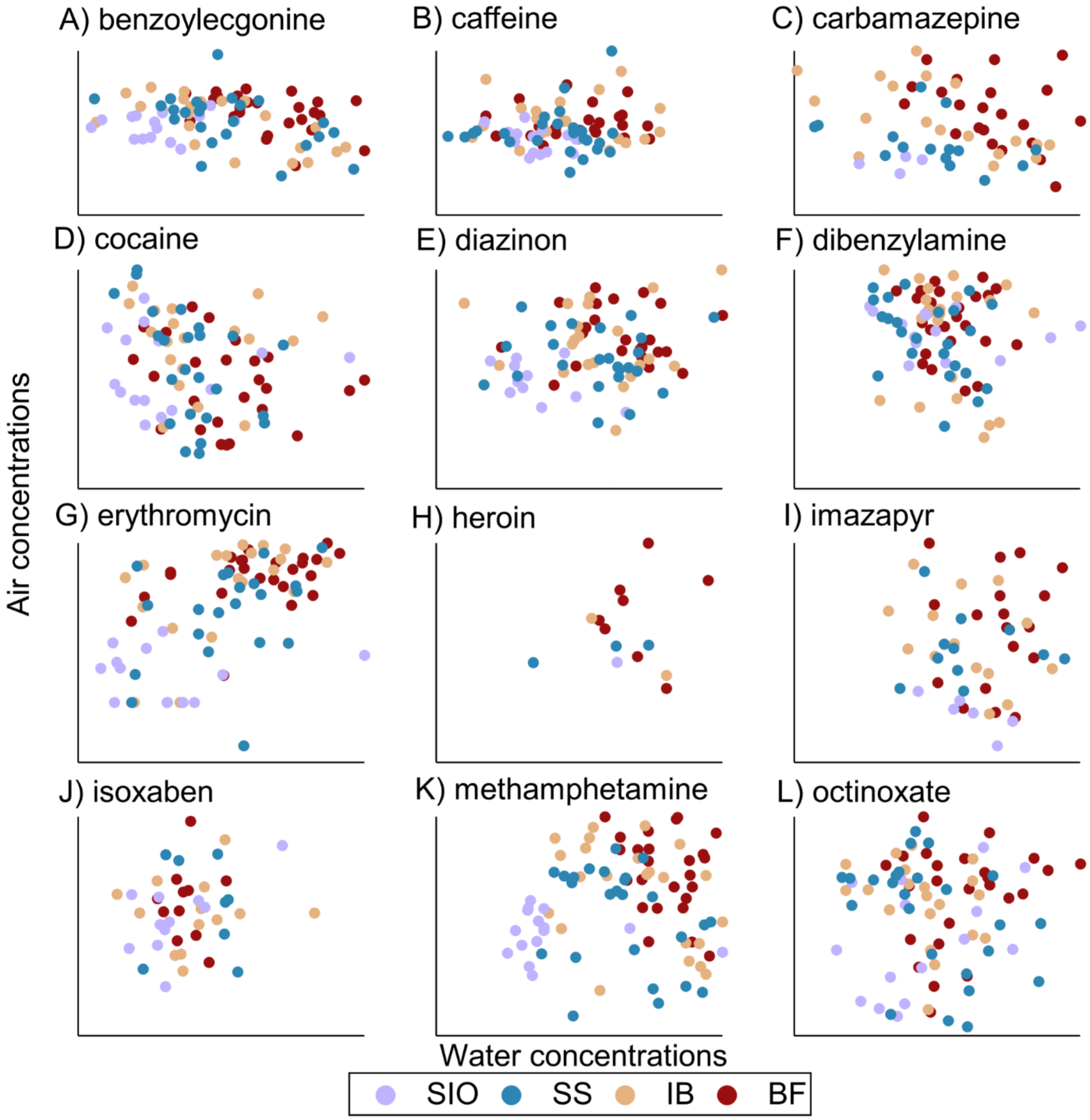

Figure S2. Co-variations in the aerosol and water concentrations for each pollutant depending on the measurement site. Data is shown for A) benzoylecgonine B) caffeine C) carbamazepine D) cocaine E) diazinon F) dibenzylamine G) erythromycin H) heroin I) imazapyr J) isoxaben K) methamphetamine and L) octinoxate. Data are from concurrent aerosol and water measurements at the Scripps Institution of Oceanography (SIO), Silver Strand (SS), Imperial Beach (IB), and Border Field State Park (BF). Data points that are more to the right and up in the plots indicate greater concentrations in both aerosol and water samples.

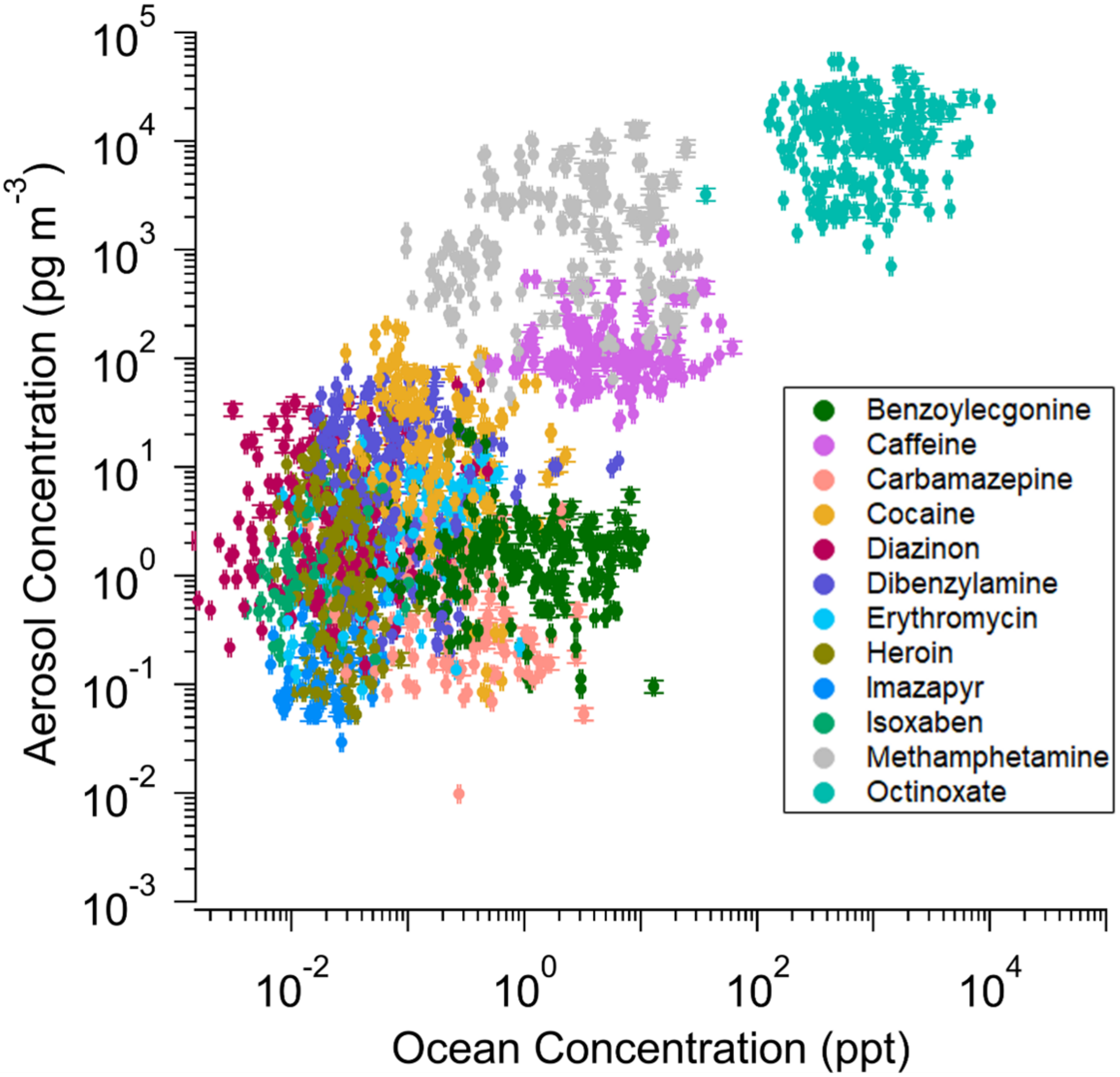

Figure S3. Concentrations of targeted wastewater chemicals in aerosol and ocean water samples at all sampling sites. Error bars represent the uncertainty in concentration propagated from the uncertainty in each chemical's sensitivity.

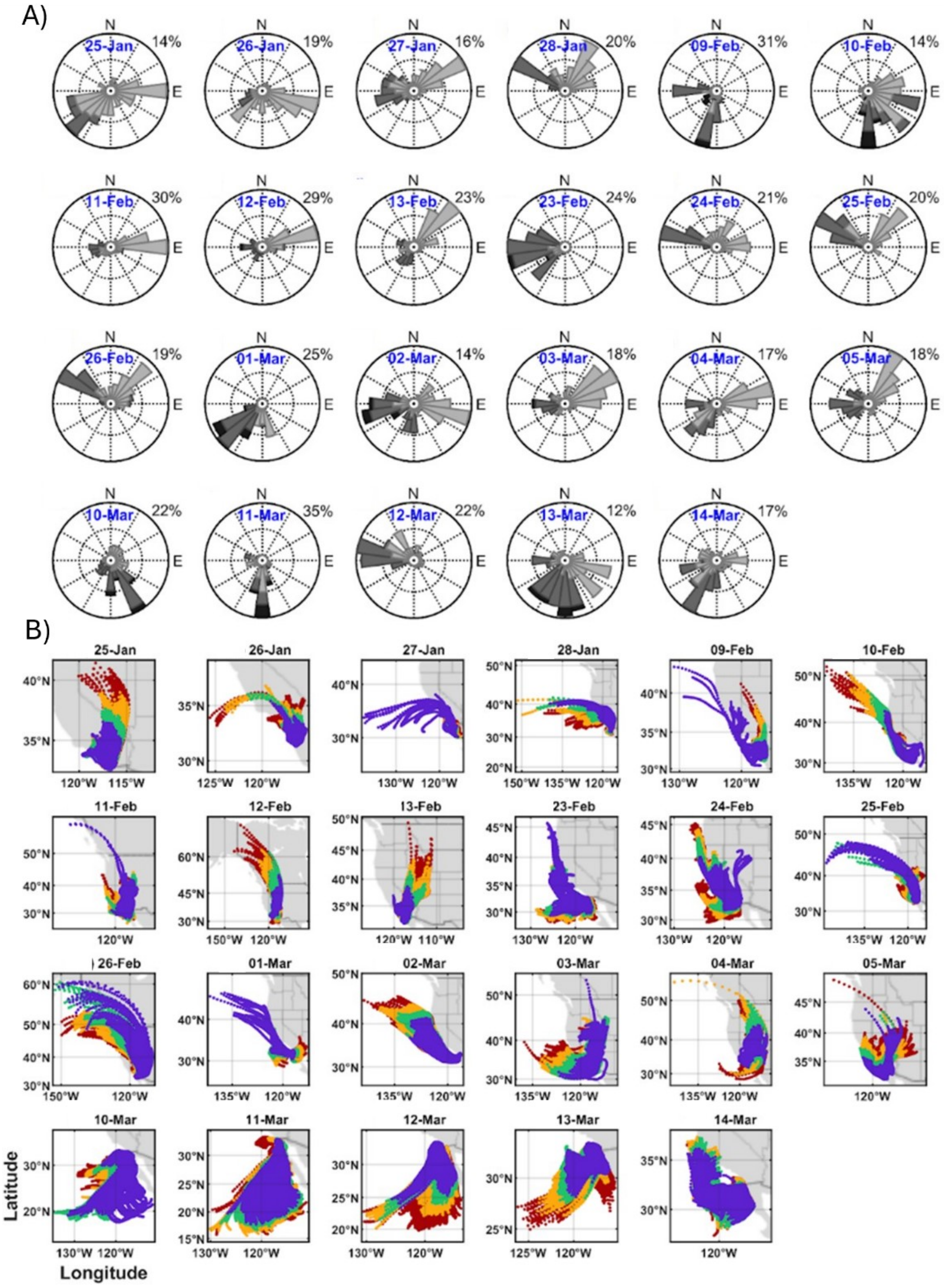

Figure S4. Local winds and back trajectories for the southern sampling locations BF, IB, and SS. Wind roses show the fraction of the sampling time wind blew from a given direction at multiple wind speed intervals: light gray: 0-2 m/s; dark gray: 2-4 m/s; black: 4+ m/s. Local winds (A) primarily show a mixture of onshore and offshore wind for each sampling period, common to coastal areas. Back trajectories (B) show individual simulated particles in four colors for the four FLEXPART runs for each sampling period. Like the local winds, the back trajectories show continental/terrestrial and marine air mass histories for each sampling period.

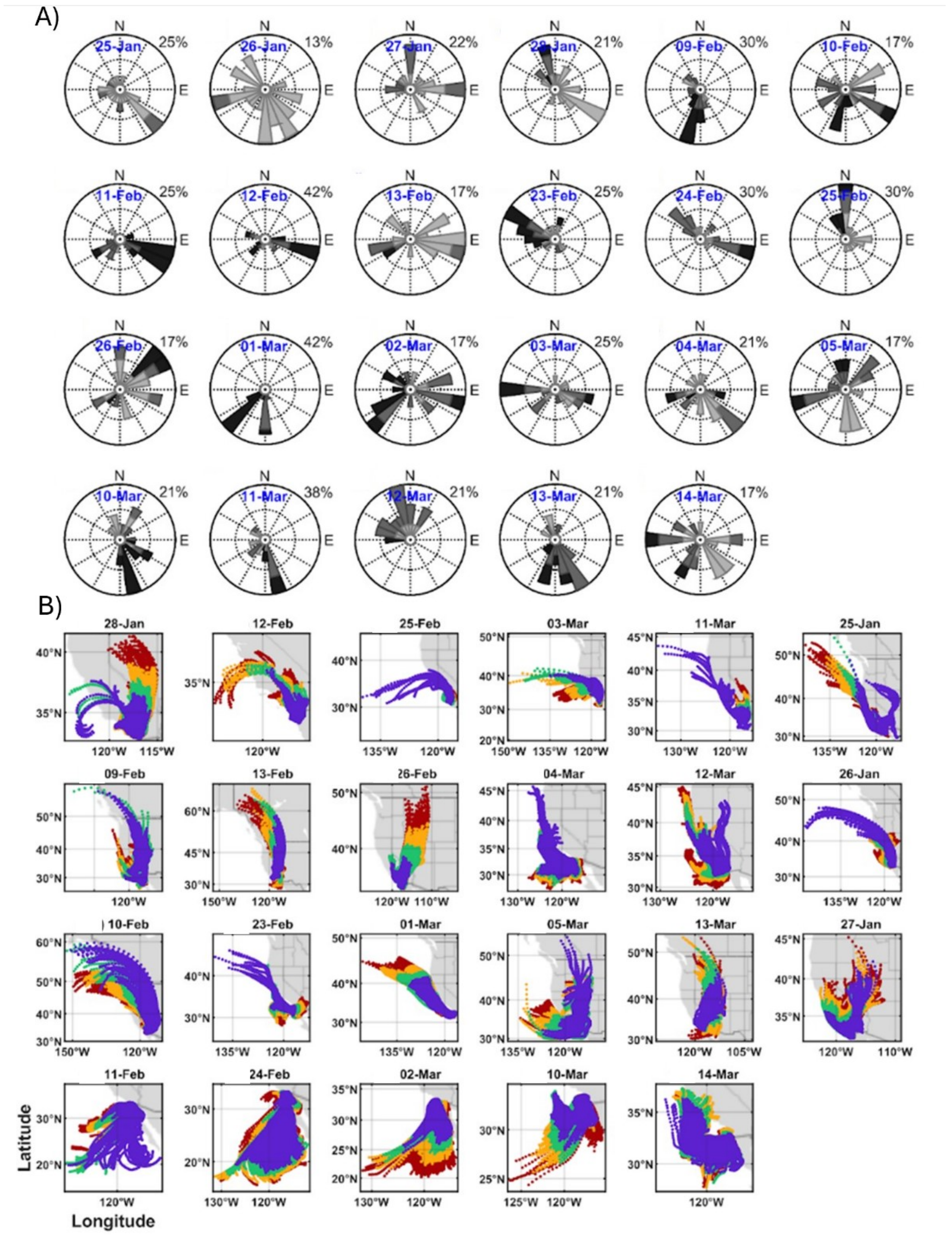

Figure S5. Local winds and back trajectories for the SIO sampling location. Wind roses show the fraction of the sampling time wind blew from a given direction at multiple wind speed intervals: light gray: 0-2 m/s; dark gray: 2-4 m/s; black: 4+ m/s. Local winds (A) primarily show a mixture of onshore and offshore wind for each sampling period, common to coastal areas. Back trajectories (B) show individual simulated particles in four colors for the four FLEXPART runs for each sampling period. Like the local winds, the back trajectories show continental/terrestrial and marine air mass histories for each sampling period.

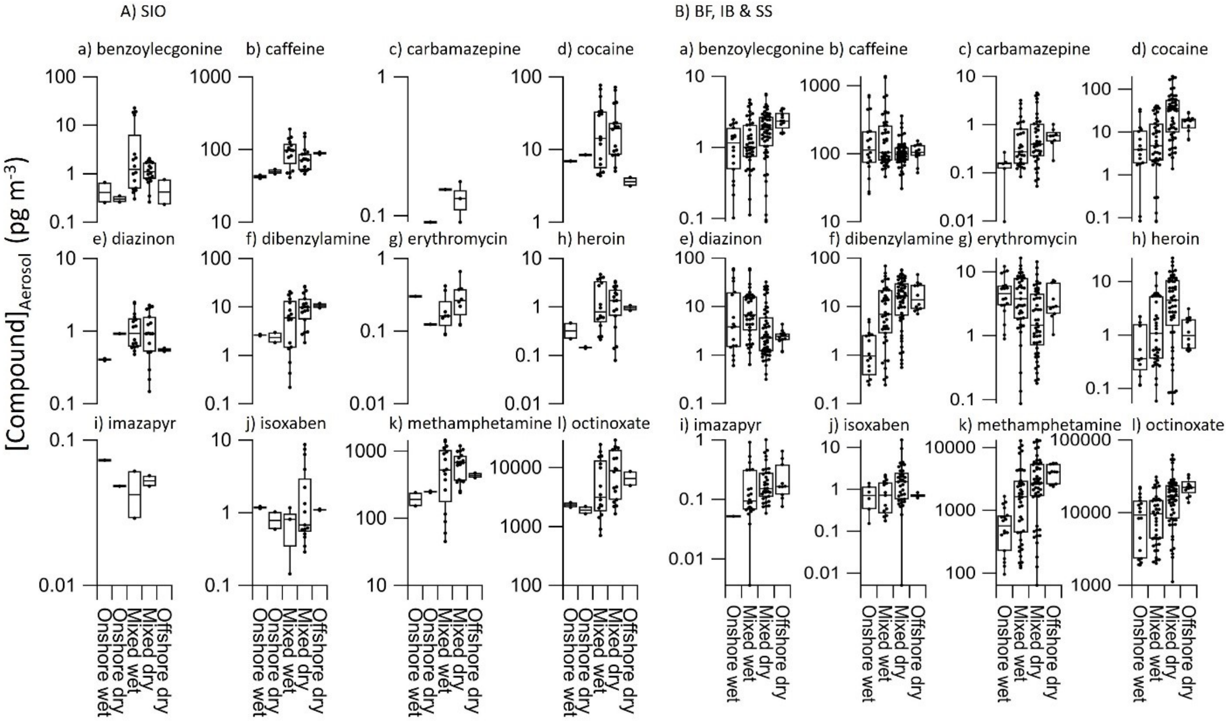

Figure S6. The influence of primary air mass origin and precipitation on aerosol contaminants. A) SIO. Samples were separated into onshore wet (n=1 sampling day,) onshore dry (n=1,) mixed wet (n=9,) mixed dry (n=8) and offshore dry (n=1.) B) BF, IB & SS. Samples were separated into onshore wet (n=3,) mixed wet (n=9,) mixed dry (n=7,) and offshore dry (n=2.) Box plots show the median as a horizontal line, the boxes indicate the 25th and 75th percentiles, and the whiskers represent the 5th and 95th percentiles.

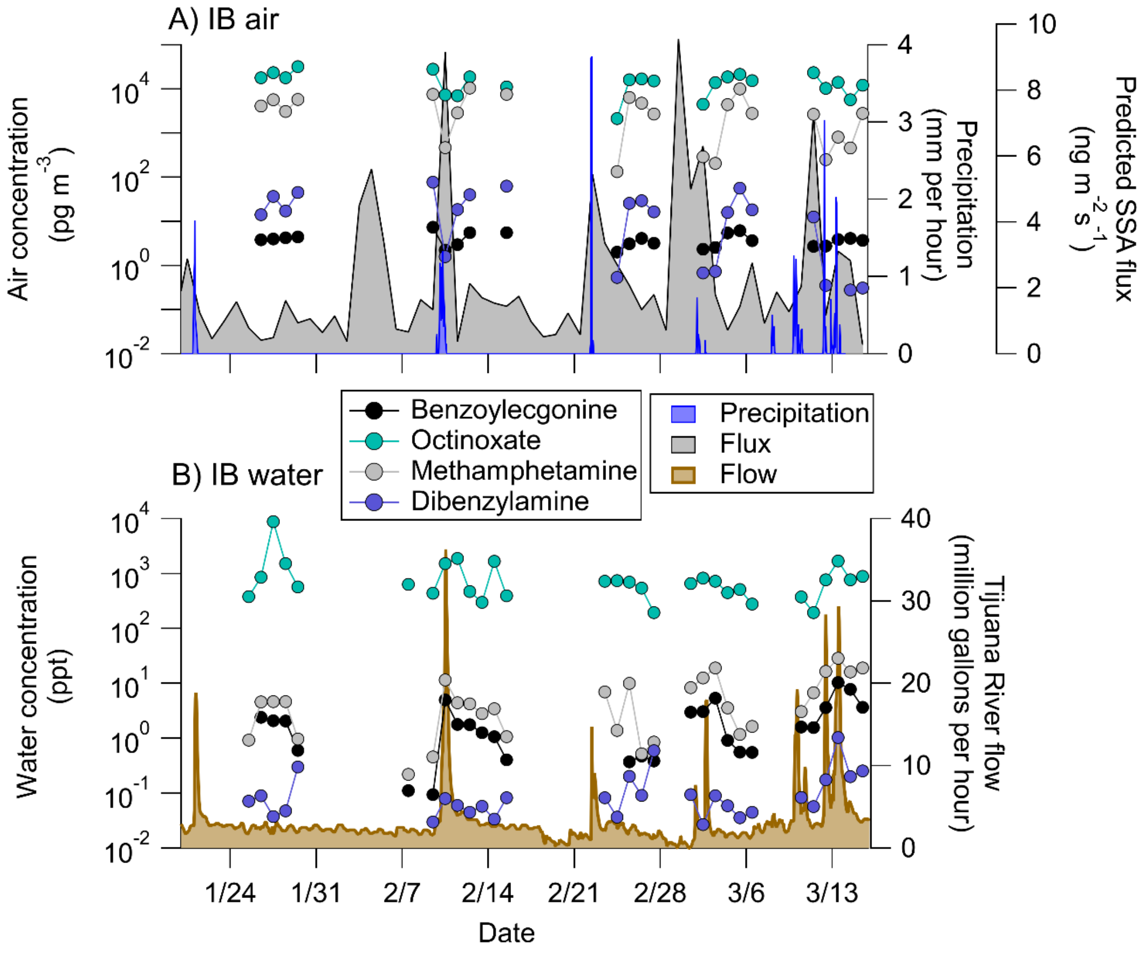

Figure S7. Temporal profiles of select pollutant concentrations in aerosol and ocean water at Imperial Beach. A) Time series of airborne pollutant concentrations suspected to undergo sea-to-air transfer via SSA (left) as well as precipitation rates and predicted SSA flux (right.) B) Time series of waterborne pollutant concentrations (left) and Tijuana River flow rate (right.)

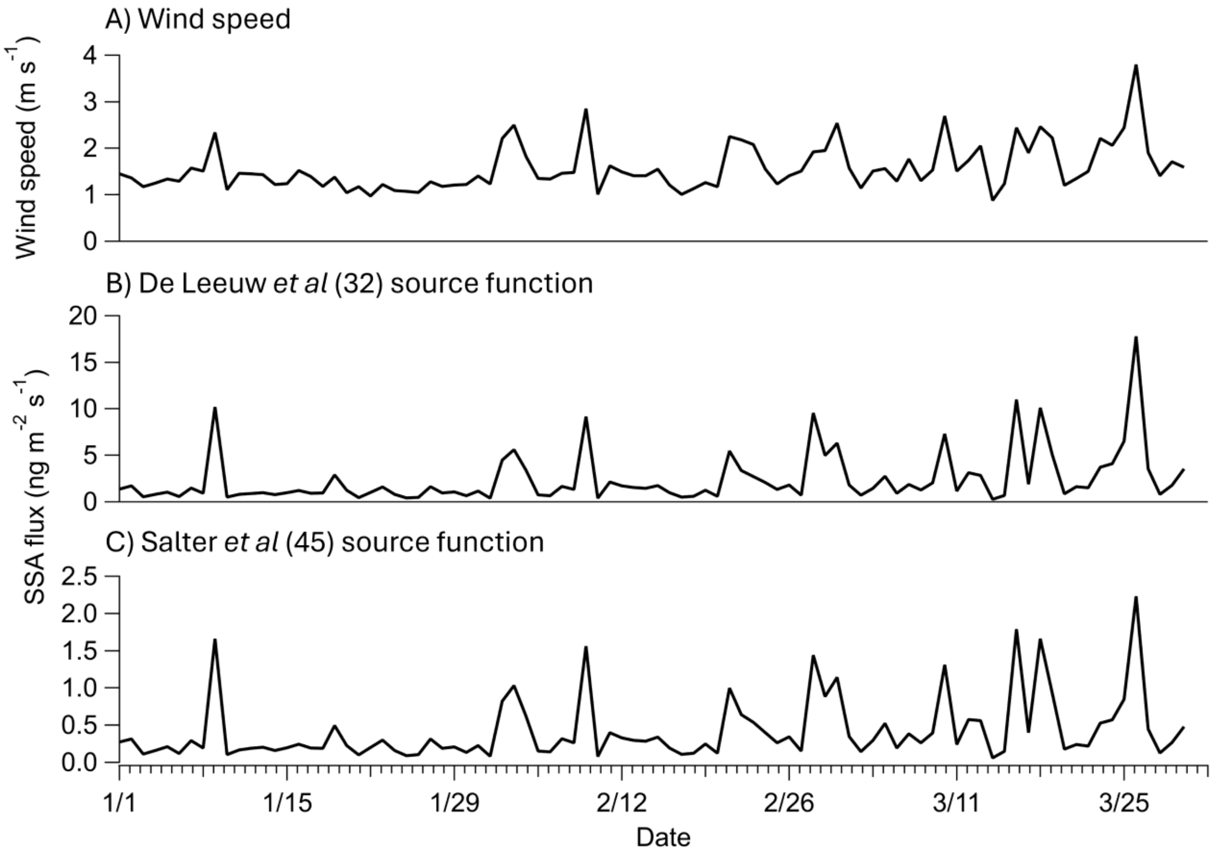

Figure S8. Comparison between different SSA source functions. A) Time series of wind speed measured by a meteorological station in IB. B) Time series of SSA flux calculated using the De Leeuw 2011 (*32*) source function. C) Time series of SSA flux calculated using the Salter 2015 (*43*) source function.

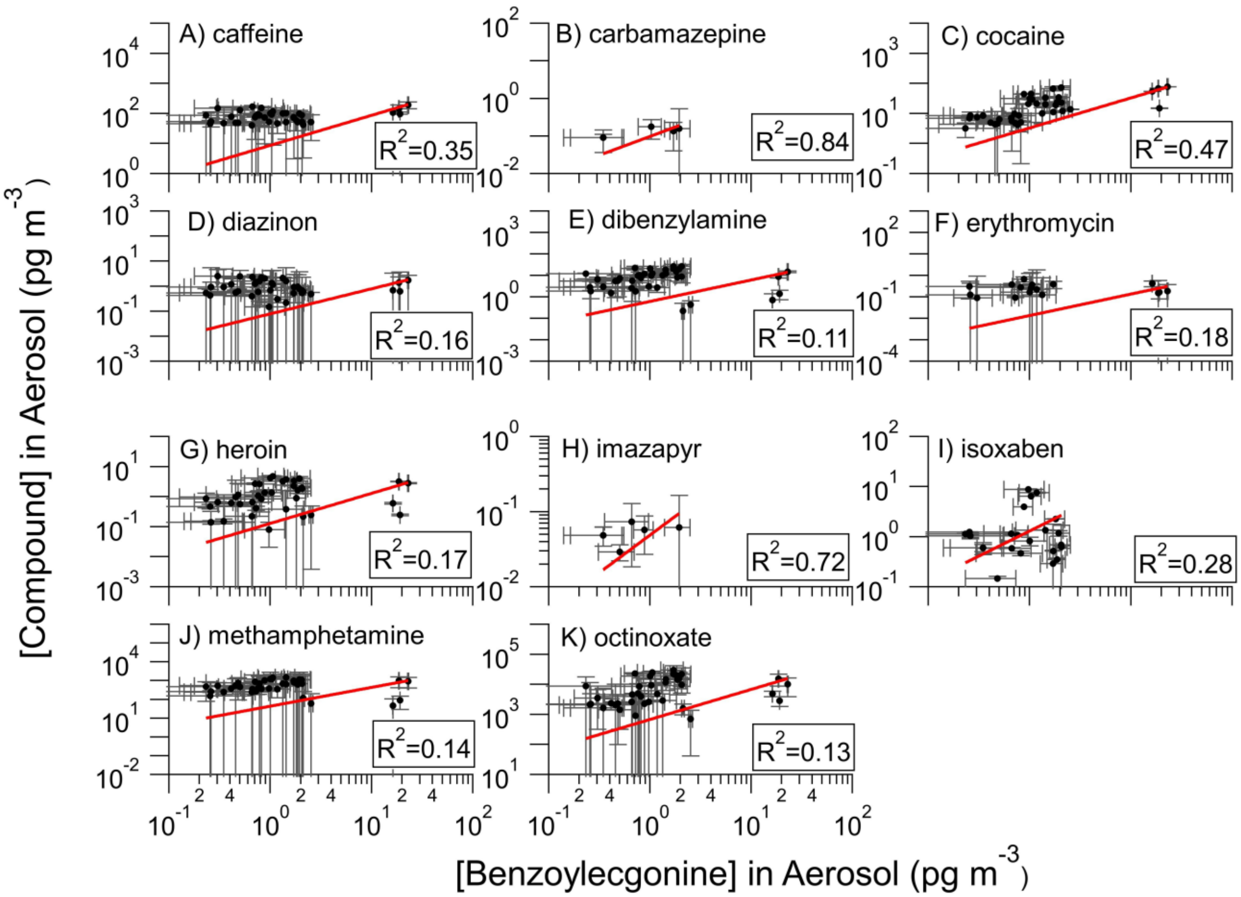

Figure S9. Linear regressions between the quantified pollutants and benzoylecgonine in aerosols from the SIO measurement site. A) caffeine, B) carbamazepine, C) cocaine, D) diazinon, E) dibenzylamine, F) erythromycin, G) heroin, H) imazapyr, I) isoxaben, J) methamphetamine, and K) octinoxate. Error bars reflect the relative uncertainty in the calibrated sensitivity for each compound. Horizontal error bars that extend to the left axis were below the quantification limit of benzoylecgonine and extended to its detection limit. Vertical error bars that extend to the bottom axis were below the quantification limit of the pollutant and extended to their detection limits. The intercept of the line of best fit is set to zero.

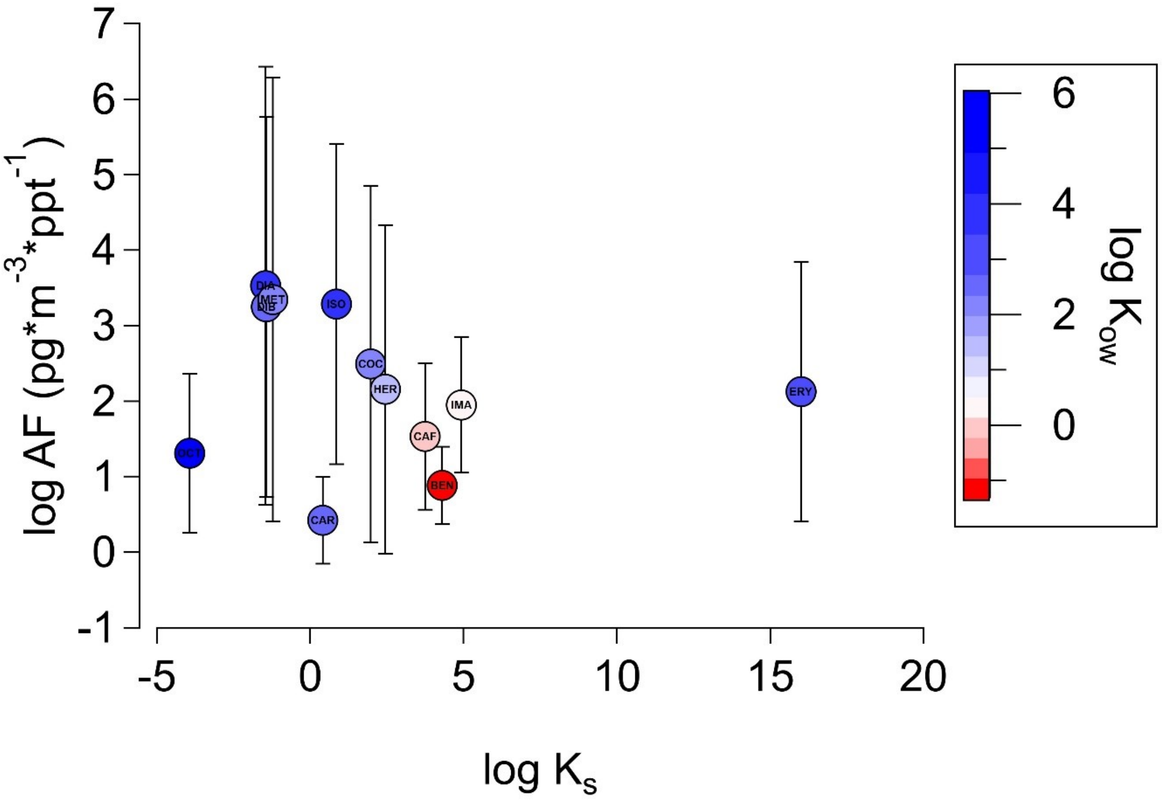

Figure S10. Average log aerosolization factors (AF) of 12 different micropollutant compounds as a function of their bubble scavenging coefficient, K_s. Log K_s values below -2.5 imply that a compound is primarily present in water during scavenging. Log K_s values between -2.5 and 2.5 imply a compound is present at the water-bubble interface. Log K_s values above 2.5 imply that a compound is primarily entrained inside the air bubble. Data points are colored by their octanol-air partitioning coefficient, K_{ow}. Only days exhibiting predominantly marine-influenced air masses at BF, IB, and SS are included.

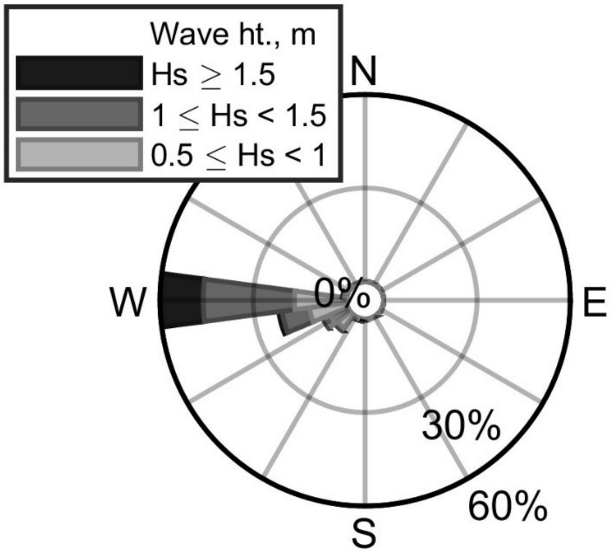

Figure S11. Local swell during the sampling periods. Swell data are shown for all sampling periods combined. Pictured is the percentage of time the swell came from a given direction with a given swell height: light gray: 0.5-1 m; dark gray: 1-1.5 m; black: 1.5+ m. The swell largely came from the west during the study period, which would not have generated strong alongshore transport. However, no transport is expected to the south, given some contribution from swell from the southwest.

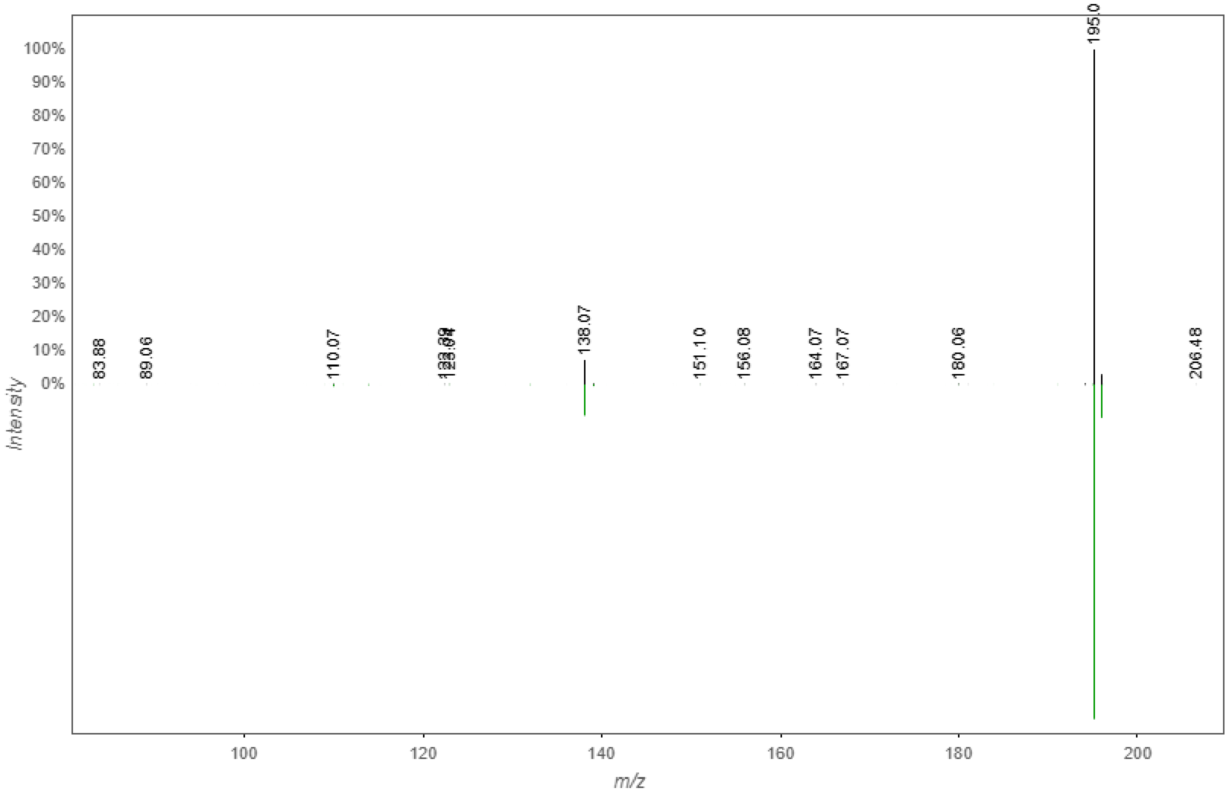

Figure S12. Mirror match between the MS2 spectra of caffeine in a sample (top) and standard (bottom).

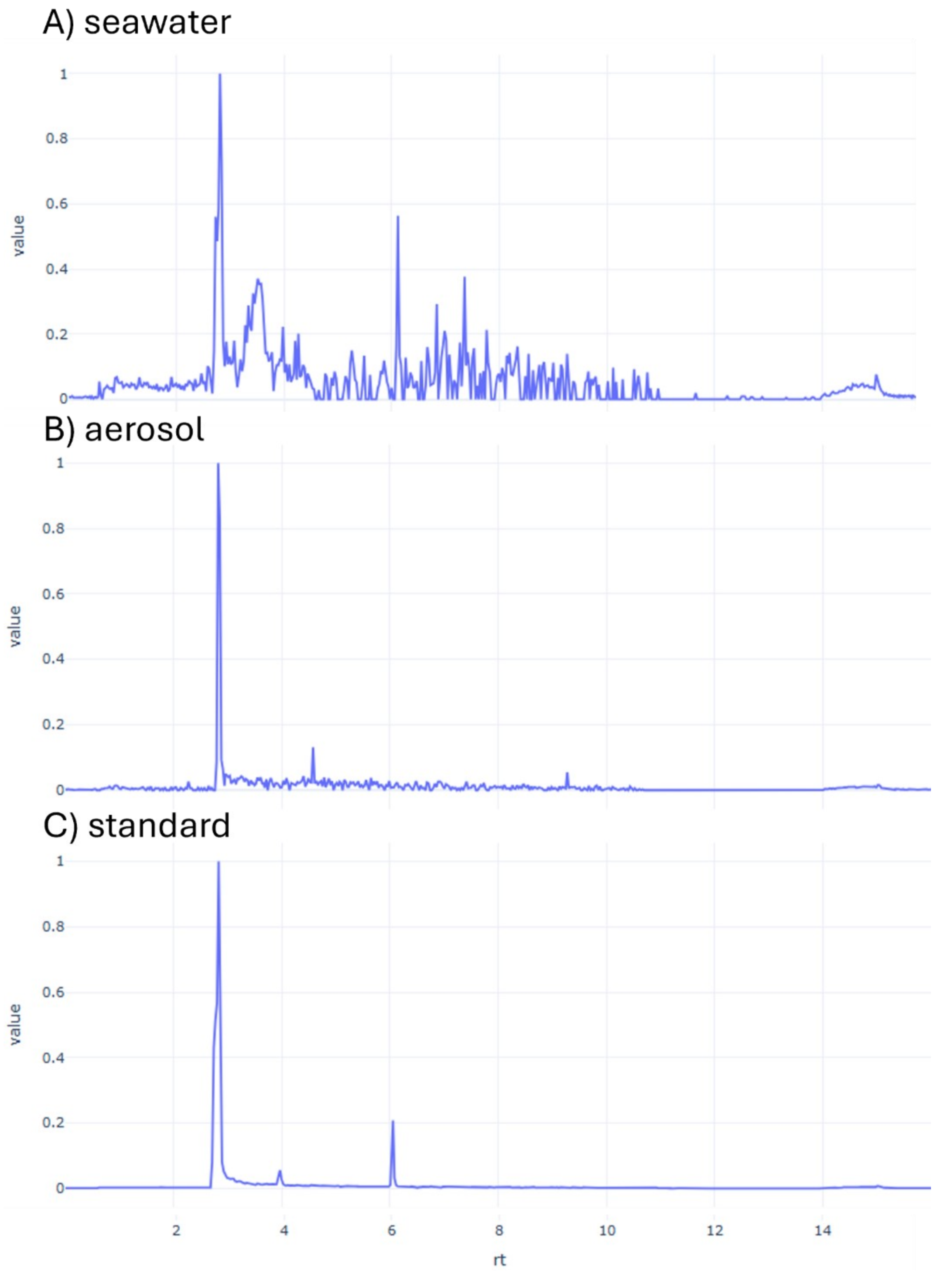

Figure S13. Representative total ion extraction plots for caffeine (m/z=195.09). A) seawater sample, B) aerosol sample (both collected at IB on 1/26) compared to a C) standard. Further filtering based on MS2 spectra is used prior to quantification.

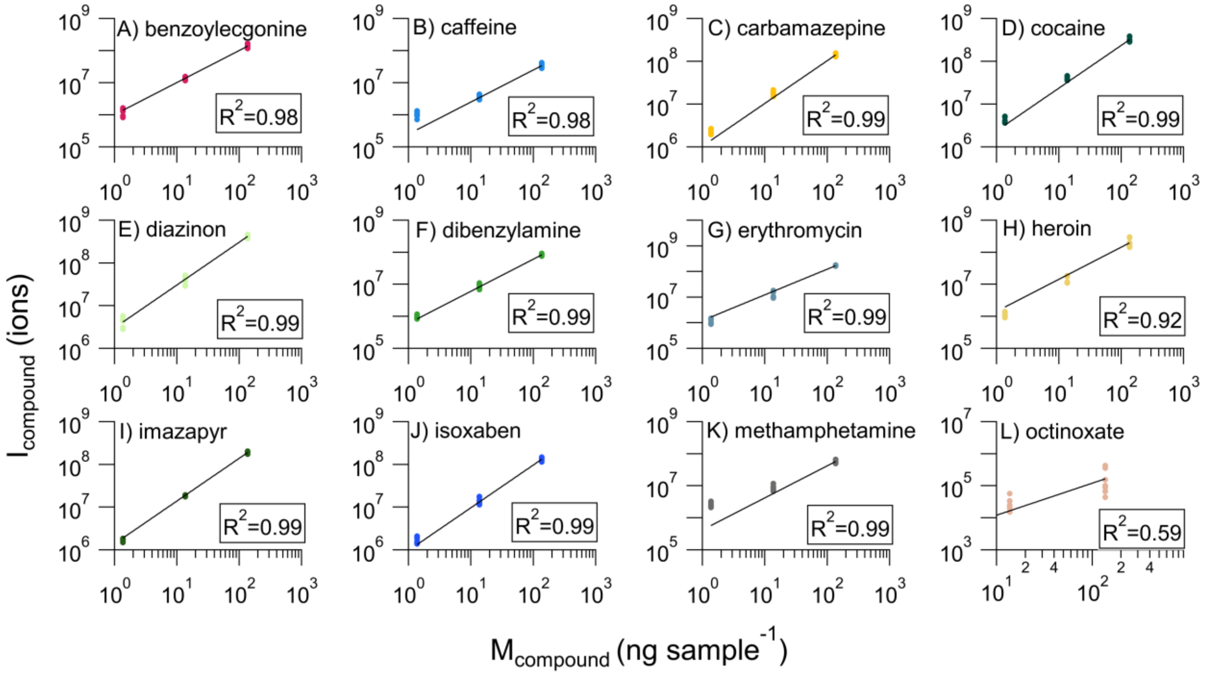

Figure S14. Calibration curves. Analytical figures of merit are given in **Table S4.** A) benzoylecgonine, B) caffeine, C) carbamazepine, D) cocaine, E) diazinon, F) dibenzylamine, G) erythromycin, H) heroin, I) imazapyr, J) isoxaben, K) methamphetamine, and L) octinoxate.

Table S1. Median concentrations in ppt "part per trillion" and detection frequencies (DF in %) for 12 organic pollutants in water samples collected at each sampling site. Locations: Tijuana River – TJR, Border Field State Park – BF, Imperial Beach – IB, Silver Strand State Park – SS, and Scripps Institution of Oceanography Pier – SIO.

Compound	Identity	Median TJR ppt (DF)	Median BF ppt (DF)	Median IB ppt (DF)	Median SS ppt (DF)	Median SIO ppt (DF)
**Benzoylecgonine**	Metabolite, Cocaine	29 (100%)	2 (100%)	1 (100%)	1 (100%)	0.26 (100%)
**Caffeine**	Stimulant	214 (100%)	8 (100%)	6 (100%)	6 (100%)	3 (100%)
**Carbamazepine**	Pharmaceutical, anticonvulsant	7.0 (100%)	0.41 (98%)	0.25 (97%)	0.183 (95%)	0.062 (100%)
**Cocaine**	Illicit Drug	0.12 (100%)	0.252 (100%)	0.15 (100%)	0.16 (100%)	0.096 (100%)
**Diazinon**	Insecticide	0.054 (86%)	0.018 (85%)	0.014 (86%)	0.021 (92%)	0.010 (75%)
**Dibenzylamine**	Materials, Rubber	0.060 (95%)	0.070 (92%)	0.073 (89%)	0.060 (85%)	0.061 (95%)
**Erythromycin**	Pharmaceutical, Antibiotic	0.27 (95%)	0.090 (85%)	0.062 (79%)	0.072 (75%)	0.019 (56%)
**Heroin**	Illicit Drug	0.051 (100%)	0.033 (96%)	0.027 (94%)	0.025 (95%)	0.025 (99%)
**Imazapyr**	Herbicide	0.180 (100%)	0.019 (80%)	0.016 (81%)	0.014 (80%)	0.015 (86%)
**Isoxaben**	Herbicide	0.032 (69%)	0.015 (47%)	0.016 (66%)	0.019 (63%)	0.010 (57%)
**Methamphetamine**	Illicit Drug	110 (100%)	5 (100%)	4 (100%)	2 (100%)	0.35 (100%)
**Octinoxate**	UV Filter	5000 (100%)	970 (100%)	650 (100%)	600 (100%)	710 (100%)

Table S2. Median concentrations in pg m^{-3} and detection frequencies (DF in %) for 12 organic pollutants in air samples collected at each sampling site. Locations: Tijuana River – TJR, Border Field State Park – BF, Imperial Beach – IB, Silver Strand State Park – SS, Scripps Institution of Oceanography Pier – SIO.

**Compound**	**Identity**	**Median BF** pg m^{-3} **(DF)**	**Median IB** pg m^{-3} **(DF)**	**Median SS** pg m^{-3} **(DF)**	**Median SIO** pg m^{-3} **(DF)**
**Benzoylecgonine**	Metabolite, Cocaine	2.0 (100%)	1.9 (100%)	1.4 (100%)	1.0 (100%)
**Caffeine**	Stimulant	110 (100%)	120 (100%)	90 (100%)	84 (100%)
**Carbamazepine**	Pharmaceutical, anticonvulsant	0.5 (81%)	0.6 (75%)	0.2 (50%)	0.1 (12.5%)
**Cocaine**	Illicit Drug	10 (100%)	28 (96%)	20 (100%)	16 (100%)
**Diazinon**	Insecticide	5.9 (100%)	2.2 (100%)	2.1 (96%)	0.78 (98%)
**Dibenzylamine**	Materials, Rubber	14 (100%)	15 (94%)	9.6 (100%)	8.4 (100%)
**Erythromycin**	Pharmaceutical, Antibiotic	3.9 (97%)	5.0 (94%)	1.9 (79%)	0.2 (40%)
**Heroin**	Illicit Drug	1.7 (100%)	5.7 (79%)	2.6 (92%)	0.9 (98%)
**Imazapyr**	Herbicide	0.1 (68%)	0.1 (65%)	0.1 (46%)	0.1 (10%)
**Isoxaben**	Herbicide	1.3 (46%)	0.7 (56%)	1.7 (50%)	1.0 (60%)
**Methamphetamine**	Illicit Drug	3000 (100%)	3100 (100%)	1800 (100%)	660 (100%)
**Octinoxate**	UV Filter	19000 (100%)	15000 (100%)	14000 (100%)	5800 (100%)

Table S3. Mann-Whitney U p-values for comparisons between locations in water samples. p<0.01 is bolded.

	TJ to SIO	BF to SIO	IB to SIO	SS to SIO
**Benzoylecgonine**	**0.000**	**0.000**	**0.000**	**0.000**
**Caffeine**	**0.000**	**0.000**	**0.000**	**0.000**
**Carbamazepine**	**0.000**	**0.000**	**0.000**	**0.000**
**Cocaine**	0.031	**0.000**	**0.000**	0.097
**Diazinon**	**0.000**	**0.000**	0.020	**0.000**
**Dibenzylamine**	0.850	0.419	0.178	0.527
**Erythromycin**	**0.000**	**0.000**	**0.000**	**0.000**
**Heroin**	**0.000**	**0.001**	0.610	**0.000**
**Imazapyr**	**0.000**	**0.009**	0.272	**0.000**
**Isoxaben**	**0.000**	**0.003**	**0.009**	**0.000**
**Methamphetamine**	**0.000**	**0.000**	**0.000**	**0.000**
**Octinoxate**	**0.000**	0.087	0.581	**0.000**

Table S4. Mann-Whitney U p-values for comparisons between locations in air samples. p<0.01 is bolded.

	BF to SIO	IB to SIO	SS to SIO
**Benzoylecgonine**	**0.001**	**0.001**	0.267
**Caffeine**	**0.000**	**0.000**	0.167
**Carbamazepine**	**0.001**	**0.001**	0.127
**Cocaine**	0.040	0.518	0.898
**Diazinon**	**0.000**	**0.000**	**0.000**
**Dibenzylamine**	0.013	0.049	0.393
**Erythromycin**	**0.000**	**0.000**	**0.000**
**Heroin**	0.146	**0.000**	**0.006**
**Imazapyr**	**0.002**	**0.001**	**0.006**
**Isoxaben**	0.428	0.131	0.288
**Methamphetamine**	**0.000**	**0.000**	**0.000**
**Octinoxate**	**0.000**	**0.000**	**0.004**

Table S5. Sampling conditions for each aerosol sample.

Sampling Date	Air Mass Influence - Southern sites	Air Mass Influence - SIO	Precipitation Conditions
**1/25/2020**	land	mixed	dry
**1/26/2020**	land	mixed	dry
**1/27/2020**	mixed	mixed	dry
**1/28/2020**	mixed	mixed	dry
**2/9/2020**	mixed	mixed	dry
**2/10/2020**	mixed	mixed	wet
**2/11/2020**	mixed	mixed	wet
**2/12/2020**	mixed	mixed	dry
**2/13/2020**	land	land	dry
**2/23/2020**	sea	sea	wet
**2/24/2020**	mixed	mixed	dry
**2/25/2020**	mixed	mixed	dry
**2/26/2020**	mixed	land	dry
**3/1/2020**	mixed	sea	dry
**3/2/2020**	mixed	mixed	wet
**3/3/2020**	mixed	mixed	wet
**3/4/2020**	mixed	mixed	dry
**3/5/2020**	mixed	mixed	dry
**3/10/2020**	mixed	mixed	wet
**3/11/2020**	mixed	mixed	wet
**3/12/2020**	sea	mixed	wet
**3/13/2020**	mixed	mixed	wet
**3/14/2020**	sea	mixed	wet

Table S6. Mann-Whitney U p-values for comparisons between air mass origin for southern locations (BF,IB & SS.) p<0.01 is bolded.

	Onshore wet - mixed wet	Offshore dry - mixed dry	Onshore wet - offshore dry
**Benzoylecgonine**	0.493	0.057	**0.000**
**Caffeine**	0.765	0.533	0.568
**Carbamazepine**	0.048	0.523	**0.007**
**Cocaine**	0.523	0.092	**0.004**
**Diazinon**	0.345	0.899	0.434
**Dibenzylamine**	**0.000**	0.901	**0.000**
**Erythromycin**	0.604	0.062	0.350
**Heroin**	0.103	**0.006**	0.103
**Imazapyr**	0.200	0.652	0.155
**Isoxaben**	0.726	0.304	0.897
**Methamphetamine**	**0.006**	0.241	**0.000**
**Octinoxate**	0.567	0.035	**0.000**

Table S7. Mann-Whitney U p-values for comparisons between air mass origin for SIO. p<0.01 is bolded. Missing values indicate the compound was not detected under those conditions.

	Onshore wet - mixed wet	Offshore dry - mixed dry	Onshore dry - Offshore dry	Onshore wet - offshore dry
Benzoylecgonine	0.140	0.089	0.699	0.699
Caffeine	**0.042**	0.284	0.245	0.245
Carbamazepine				
Cocaine	0.440	0.027	0.245	0.245
Diazinon	**0.029**	0.388	0.245	0.245
Dibenzylamine	0.623	0.753	0.245	0.245
Erythromycin	0.561			
Heroin	0.140	0.642	0.245	0.245
Imazapyr	0.540			
Isoxaben	0.386	0.709	0.245	0.245
Methamphetamine	0.292	0.488	0.245	0.245
Octinoxate	0.623	0.950	0.245	0.245

Table S8. Physical parameters for compounds used in aerosolization factor (AF) analysis. K_{ow}, saturation vapor pressure, and Henry's Law constant are from the EPA EPI Suite from experimental databases or modeled estimates.(*98*) Ka is calculated as RT**p*$^{-1}$. Kaq is calculated as H^{-1}**p*$^{-1}$.

Compound	log K_{ow}	Saturation vapor pressure (*p*) (Pa)	Henry's Law constant (H) (Pa*m^{-3}*mol)	log K_a	log K_{aq}
**Benzoylecgonine**	-1.32	1.1E-07	2.0E-08	14.64	10.35
**Caffeine**	-0.07	9.8E-07	7.2E-08	13.15	9.40
**Carbamazepine**	2.45	1.2E-05	1.6E-04	8.74	8.33
**Cocaine**	2.30	2.6E-05	4.3E-06	9.96	7.99
**Diazinon**	3.81	1.2E-02	1.1E-02	3.86	5.32
**Dibenzylamine**	2.67	7.9E-04	1.1E-02	5.07	6.50
**Erythromycin**	3.06	2.8E-23	4.0E-20	41.94	25.94
**Heroin**	1.58	1.0E-07	1.4E-06	12.84	10.39
**Imazapyr**	0.22	1.2E-08	4.8E-09	16.24	11.32
**Isoxaben**	3.94	5.5E-07	5.6E-05	10.51	9.65
**Methamphetamine**	2.07	6.0E-01	6.7E-03	2.40	3.62
**Octinoxate**	5.80	1.9E-03	3.5E+00	2.19	6.13

Table S9. List of quantified micropollutant compounds and their classes along with figures of merit. Included values: calibration curve R^2, sensitivity to sample mass, instrumental limits of detection (LOD) for water (left columns) and aerosol samples (right columns), and calibration LOD for the same. The signal-to-noise ratio determined instrumental LOD. Field and method blank measurements determined method LOD. The external calibration curve determined calibration LOD. See **Figure S10** for plots of calibration curves.

Compound	Class	Calibration Curve R^2	Sensitivity (RSD) (ions ng^{-1}) (%)	Instrument LOD		Method LOD		Calibration LOD	
**Benzoylecgonine**	Metabolite, Cocaine	0.98	1.0E+06 (3%)	1.2 ppq	20 fg*m^{-3}	8.1 ppq	140 fg*m^{-3}	120 ppq	1.9 pg*m^{-3}
**Caffeine**	Stimulant	0.98	2.5E+05 (3%)	4.9 ppq	81 fg*m^{-3}	8.0 ppt	130 pg*m^{-3}	130 ppq	2.2 pg*m^{-3}
**Carbamazepine**	Pharmaceutical ,anticonvulsant	1.00	1.0E+06 (1%)	1.2 ppq	20 fg*m^{-3}	7.0 ppq	120 fg*m^{-3}	52 ppq	870 fg*m^{-3}
**Cocaine**	Illicit Drug	0.99	2.3E+06 (3%)	0.51 ppq	8.5 fg*m^{-3}	11 ppq	180 fg*m^{-3}	100 ppq	1.7 pg*m^{-3}
**Diazinon**	Insecticide	0.99	3.0E+06 (2%)	0.40 ppq	6.6 fg*m^{-3}	860 ppq	14 pg*m^{-3}	67 ppq	1.1 pg*m^{-3}
**Dibenzylamine**	Materials, Rubber	0.99	6.0E+05 (2%)	2.0 ppq	33 fg*m^{-3}	320 ppq	5.3 pg*m^{-3}	61 ppq	1.0 pg*m^{-3}
**Erythromycin**	Pharmaceutical , Antibiotic	1.00	1.2E+06 (1%)	1.0 ppq	17 fg*m^{-3}	14 ppq	230 fg*m^{-3}	64 ppq	1.1 pg*m^{-3}
**Heroin**	Illicit Drug	0.92	1.4E+06 (6%)	0.85 ppq	14 fg*m^{-3}	1.9 ppq	31 fg*m^{-3}	240 ppq	17 pg*m^{-3}

**Imazapyr**	Herbicide	1.00	1.4E+06 (1%)	0.87 ppq	14 fg*m^{-3}	3.0 ppq	50 fg*m^{-3}	49 ppq	820 fg*m^{-3}
**Isoxaben**	Herbicide	0.99	9.6E+05 (1%)	1.3 ppq	21 fg*m^{-3}	1.3 ppq	5.2 fg*m^{-3}	60 ppq	1.0 pg*m^{-3}
**Methamphetamine**	Illicit Drug	0.99	4.2E+05 (2%)	2.9 ppq	48 fg*m^{-3}	1.2 ppt	19 pg*m^{-3}	82 ppq	1.4 pg*m^{-3}
**Octinoxate**	UV Filter	0.59	1.2E+03 (21%)	1.0 ppt	17 pg*m^{-3}	56 ppt	930 pg*m^{-3}	1.0 ppt	17 ng*m^{-3}

REFERENCES AND NOTES

1. J. Svejkovsky, N. P. Nezlin, N. M. Mustain, J. B. Kum, Tracking stormwater discharge plumes and water quality of the Tijuana River with multispectral aerial imagery. *Estuar. Coast. Shelf Sci.* **87**, 387–398 (2010).

2. L. Fernandez, Wastewater pollution abatement across an international border. *Environ. Dev. Econ.* **14**, 67–88 (2009).

3. B. Rico, K. C. Barsanti, W. C. Porter, P. Stigler-Granados, K. A. Prather, Heavily polluted Tijuana River drives regional air quality crisis. ChemRxiv (2024). http://doi.org/10.26434/CHEMRXIV-2024-MJGBR.

4. F. Feddersen, A. B. Boehm, S. N. Giddings, X. Wu, D. Liden, Modeling untreated wastewater evolution and swimmer illness for four wastewater infrastructure scenarios in the San Diego-Tijuana (US/MX) border region. *Geohealth* **5**, e2021GH000490 (2021).

5. County of San Diego Board of Supervisors, FRAMEWORK FOR OUR FUTURE: DECLARING POLLUTION AT THE TIJUANA RIVER VALLEY A PUBLIC HEALTH CRISIS (DISTRICTS: ALL). 1–2 (2021).

6. United States Environmental Protection Agency, Joint Record of Decision for the Final Programmatic Environmental Impact Statement for United States-Mexico-Canada Agreement Mitigation of Contaminated Transboundary Flows Project. (2023).

7. M. K. Yadav, M. D. Short, R. Aryal, C. Gerber, B. Van Den Akker, C. P. Saint, Occurrence of illicit drugs in water and wastewater and their removal during wastewater treatment. *Water Res.* **124**, 713–727 (2017).

8. N. Bolong, A. F. Ismail, M. R. Salim, T. Matsuura, A review of the effects of emerging contaminants in wastewater and options for their removal. *Desalination* **239**, 229–246 (2009).

9. X. Yuan, J. Hu, S. Li, M. Yu, Occurrence, fate, and mass balance of selected pharmaceutical and personal care products (PPCPs) in an urbanized river. *Environ. Pollut.* **266**, 115340 (2020).

10. W.-R. Liu, Y.-Y. Yang, Y.-S. Liu, L.-J. Zhang, J.-L. Zhao, Q.-Q. Zhang, M. Zhang, J.-N. Zhang, Y.-X. Jiang, G.-G. Ying, Biocides in wastewater treatment plants: Mass balance analysis and pollution load estimation. *J. Hazard. Mater.* **329**, 310–320 (2017).

11. M. M. P. Tsui, H. W. Leung, T. C. Wai, N. Yamashita, S. Taniyasu, W. Liu, P. K. S. Lam, M. B. Murphy, Occurrence, distribution and ecological risk assessment of multiple classes of UV filters in surface waters from different countries. *Water Res.* **67**, 55–65 (2014).

12. A. Gogoi, P. Mazumder, V. K. Tyagi, G. G. Tushara Chaminda, A. K. An, M. Kumar, Occurrence and fate of emerging contaminants in water environment: A review. *Groundw. Sustain. Dev.* **6**, 169–180 (2018).

13. L. Fu, J. Li, G. Wang, Y. Luan, W. Dai, Ecotoxicology and Environmental Safety Adsorption behavior of organic pollutants on microplastics. *Ecotoxicol. Environ. Saf.* **217**, 112207 (2021).

14. L. Saldana, Tijuana's Toxic Waters, *North American Congress on Latin America* (2007). https://nacla.org/article/tijuana%27s-toxic-waters.

15. M. A. Pendergraft, D. J. Grimes, S. N. Giddings, F. Feddersen, C. M. Beall, C. Lee, M. V. Santander, K. A. Prather, Airborne transmission pathway for coastal water pollution. *PeerJ* **9**, e11358 (2021).

16. D. Allen, S. Allen, S. Abbasi, A. Baker, M. Bergmann, J. Brahney, T. Butler, R. A. Duce, S. Eckhardt, N. Evangeliou, T. Jickells, M. Kanakidou, P. Kershaw, P. Laj, J. Levermore, D. Li, P. Liss, K. Liu, N. Mahowald, P. Masque, D. Materić, A. G. Mayes, P. McGinnity, I. Osvath, K. A. Prather, J. M. Prospero, L. E. Revell, S. G. Sander, W. J. Shim, J. Slade, A. Stein, O. Tarasova, S. Wright, Microplastics and nanoplastics in the marine-atmosphere environment. *Nat. Rev. Earth Environ.* **3**, 393–405 (2022).

17. R. R. C. Cuadrat, M. Sorokina, B. G. Andrade, T. Goris, A. M. R. Dávila, Global ocean resistome revealed: Exploring antibiotic resistance gene abundance and distribution in TARA Oceans samples. *Gigascience* **9**, gia046 (2020).

18. C. N. Pegoraro, T. Harner, K. Su, L. Ahrens, Occurrence and Gas–Particle Partitioning of Organic UV-Filters in Urban Air. *Environ. Sci. Technol* **54**, 12881–12889 (2020).

19. A. Cecinato, C. Balducci, G. Nervegna, Occurrence of cocaine in the air of the World's cities. An emerging problem? A new tool to investigate the social incidence of drugs? *Sci. Total Environ.* **407**, 1683–1690 (2009).

20. J. Park, J. Jang, Y. J. Yoon, S. Kang, H. Kang, K. Park, K. H. Cho, J.-H. Kim, M. Dall'Osto, B. Y. Lee, When river water meets seawater: Insights into primary marine aerosol production. *Sci. Total Environ.* **807**, 150866 (2022).

21. N. W. May, J. L. Axson, A. Watson, K. A. Pratt, A. P. Ault, Lake spray aerosol generation: A method for producing representative particles from freshwater wave breaking. *Atmos. Meas. Tech.* **9**, 4311–4325 (2016).

22. P. Stigler Granados, K. Sant, P. Quintana, E. Hoh, E. Oren, N. Lopez-Galvez, M. Perez, Y. NI, Tijuana River Contamination from Urban Runoff and Sewage: A Public Health Crisis at the Border. *San Diego State University School of Public Health* (2024).

23. B. Sha, J. H. Johansson, P. Tunved, P. Bohlin-Nizzetto, I. T. Cousins, M. E. Salter, Sea spray aerosol (SSA) as a source of perfluoroalkyl acids (PFAAs) to the atmosphere: Field evidence from long-term air monitoring. *Environ. Sci. Technol.* **56**, 228–238 (2022).

24. B. Sha, J. H. Johansson, M. E. Salter, S. M. Blichner, I. T. Cousins, Constraining global transport of perfluoroalkyl acids on sea spray aerosol using field measurements. *Sci. Adv.* **10**, eadl1026 (2024).

25. S. Allen, D. Allen, K. Moss, G. Le Roux, V. R. Phoenix, J. E. Sonke, Examination of the ocean as a source for atmospheric microplastics. *PLOS ONE* **15**, e0232746 (2020).

26. C. Harb, N. Pokhrel, H. Foroutan, Quantification of the emission of atmospheric microplastics and nanoplastics via sea spray. *Environ. Sci. Technol. Lett.* **10**, 513–519 (2023).

27. B. Sha, E. Ungerovich, M. E. Salter, I. T. Cousins, J. H. Johansson, Enrichment of perfluoroalkyl acids on sea spray aerosol in laboratory experiments: The role of dissolved

organic matter, air entrainment rate and inorganic ion composition. *Environ. Sci. Technol. Lett.* **11**, 746–751 (2024).

28. E. B. Franklin, S. Amiri, D. Crocker, C. Morris, K. Mayer, J. S. Sauer, R. J. Weber, C. Lee, F. Malfatti, C. D. Cappa, T. H. Bertram, K. A. Prather, A. H. Goldstein, Anthropogenic and biogenic contributions to the organic composition of coastal submicron sea spray aerosol. *Environ. Sci. Technol.* **56**, 16633–16642 (2022).

29. T. H. Bertram, R. E. Cochran, V. H. Grassian, E. A. Stone, Sea spray aerosol chemical composition: Elemental and molecular mimics for laboratory studies of heterogeneous and multiphase reactions. *Chem. Soc. Rev.* **47**, 2374–2400 (2018).

30. P. K. Quinn, D. B. Collins, V. H. Grassian, K. A. Prather, T. S. Bates, Chemistry and related properties of freshly emitted sea spray aerosol. *Chem. Rev.* **115**, 4383–4399 (2015).

31. L. M. Russell, L. N. Hawkins, A. A. Frossard, P. K. Quinn, T. S. Bates, Carbohydrate-like composition of submicron atmospheric particles and their production from ocean bubble bursting. *Proc. Natl. Acad. Sci. U.S.A.* **107**, 6652–6657 (2010).

32. G. De Leeuw, E. L. Andreas, M. D. Anguelova, C. W. Fairall, E. R. Lewis, C. O'Dowd, M. Schulz, S. E. Schwartz, Production flux of sea spray aerosol. *Rev. Geophys.* **49**, 1–39 (2011).

33. M. Trainic, J. M. Flores, I. Pinkas, M. L. Pedrotti, F. Lombard, G. Bourdin, G. Gorsky, E. Boss, Y. Rudich, A. Vardi, I. Koren, Airborne microplastic particles detected in the remote marine atmosphere. *Commun. Earth Environ.* **1**, 64 (2020).

34. J. H. Johansson, M. E. Salter, J. C. Acosta Navarro, C. Leck, E. D. Nilsson, I. T. Cousins, Global transport of perfluoroalkyl acids via sea spray aerosol. *Environ. Sci. Process Impacts* **21**, 635–649 (2019).

35. M. A. Pendergraft, P. Beldá-Ferre, D. Petras, C. K. Morris, A. T. Aron, M. Bryant, T. Schwartz, G. Ackerman, G. Humphrey, E. Kaandorp, P. C. Dorrestein, R. Knight, K. A. Prather, Bacterial and chemical evidence of coastal water pollution from the Tijuana River in sea spray aerosol. *Environ. Sci. Technol.* **57**, 4071–4081 (2023).

36. J. Li, J. Gao, P. K. Thai, X. Sun, J. F. Mueller, Z. Yuan, G. Jiang, Stability of illicit drugs as biomarkers in sewers: From lab to reality. *Environ. Sci. Technol.* **52**, 1561–1570 (2018).

37. R. Baselt, Disposition of toxic drugs and chemicals in man, seventh edition. Biomedical Publications. *Clin. Chem.* **51**, 680 (2005).

38. X. Domènech, J. Peral, I. Muñoz, Predicted environmental concentrations of cocaine and benzoylecgonine in a model environmental system. *Water Res.* **43**, 5236–5242 (2009).

39. L. Bijlsma, C. Boix, W. M. A. Niessen, M. Ibáñez, J. V. Sancho, F. Hernández, Investigation of degradation products of cocaine and benzoylecgonine in the aquatic environment. *Sci. Total Environ.* **443**, 200–208 (2013).

40. E. Zuccato, C. Chiabrando, S. Castiglioni, D. Calamari, R. Bagnati, S. Schiarea, R. Fanelli, Cocaine in surface waters: A new evidence-based tool to monitor community drug abuse. *Environ. Health* **4**, 14 (2005).

41. G. De Leeuw, F. P. Neele, M. Hill, M. H. Smith, E. Vignati, Production of sea spray aerosol in the surf zone. *J. Geophys. Res. Atmos.* **105**, 29397–29409 (2000).

42. H. Grythe, J. Ström, R. Krejci, P. Quinn, A. Stohl, A review of sea-spray aerosol source functions using a large global set of sea salt aerosol concentration measurements. *Atmos. Chem. Phys.* **14**, 1277–1297 (2014).

43. M. E. Salter, P. Zieger, J. C. Acosta Navarro, H. Grythe, A. Kirkeväg, B. Rosati, I. Riipinen, E. D. Nilsson, An empirically derived inorganic sea spray source function incorporating sea surface temperature. *Atmos. Chem. Phys.* **15**, 11047–11066 (2015).

44. M. Lou, S. Liu, C. Gu, H. Hu, Z. Tang, Y. Zhang, C. Xu, F. Li, The bioaerosols emitted from toilet and wastewater treatment plant: A literature review. *Environ. Sci. Pollut. Res.* **28**, 2509–2521 (2021).

45. J. Socorro, A. Durand, B. Temime-roussel, S. Gligorovski, H. Wortham, E. Quivet, The persistence of pesticides in atmospheric particulate phase : An emerging air quality issue. *Nature Publishing Group* **6**, (2016).

46. I. J. Buerge, T. Poiger, M. D. Müller, H. R. Buser, Caffeine, an anthropogenic marker for wastewater contamination of surface waters. *Environ. Sci. Technol.* **37**, 691–700 (2003).

47. N. Triesch, M. Van Pinxteren, S. Frka, C. Stolle, T. Spranger, E. H. Hoffmann, X. Gong, H. Wex, D. Schulz-Bull, B. Gašparović, H. Herrmann, Concerted measurements of lipids in seawater and on submicrometer aerosol particles at the Cabo Verde islands: Biogenic sources, selective transfer and high enrichments. *Atmos. Chem. Phys* **21**, 4267–4283 (2021).

48. N. E. Olson, M. E. Cooke, J. H. Shi, J. A. Birbeck, J. A. Westrick, A. P. Ault, Harmful algal bloom toxins in aerosol generated from inland lake water. *Environ. Sci. Technol.* **54**, 4769–4780 (2020).

49. K. A. Prather, T. H. Bertram, V. H. Grassian, G. B. Deane, M. D. Stokes, P. J. DeMott, L. I. Aluwihare, B. P. Palenik, F. Azam, J. H. Seinfeld, R. C. Moffet, M. J. Molina, C. D. Cappa, F. M. Geiger, G. C. Roberts, L. M. Russell, A. P. Ault, J. Baltrusaitis, D. B. Collins, C. E. Corrigan, L. A. Cuadra-Rodriguez, C. J. Ebben, S. D. Forestieri, T. L. Guasco, S. P. Hersey, M. J. Kim, W. F. Lambert, R. L. Modini, W. Mui, B. E. Pedler, M. J. Ruppel, O. S. Ryder, N. G. Schoepp, R. C. Sullivan, D. Zhao, Bringing the ocean into the laboratory to probe the chemical complexity of sea spray aerosol. *Proc. Natl. Acad. Sci. U.S.A.* **110**, 7550–7555 (2013).

50. A. Finizio, D. Mackay, T. Bidleman, T. Harner, Octanol-air partition coefficient as a predictor of partitioning of semi-volatile organic chemicals to aerosols. *Atmos. Environ.* **31**, 2289–2296 (1997).

51. N. Sareen, S. G. Moussa, V. F. McNeill, Photochemical aging of light-absorbing secondary organic aerosol material. *J. Phys. Chem. A* **117**, 2987–2996 (2013).

52. A. Cooper, A. Shenkiryk, H. Chin, M. Morris, L. Mehndiratta, K. Roundtree, T. Tafuri, J. H. Slade, Photoinitiated degradation kinetics of the organic UV filter oxybenzone in solutions and aerosols: Impacts of salt, photosensitizers, and the medium. *ACS EST Air* **1**, 1430–1441 (2024).

53. S. M. Kruse, J. H. Slade, Heterogeneous and photosensitized oxidative degradation kinetics of the plastic additive bisphenol-A in sea spray aerosol mimics. *J. Phys. Chem. A* **127**, 4724–4733 (2023).

54. A. L. Bondy, B. Wang, A. Laskin, R. L. Craig, M. V. Nhliziyo, S. B. Bertman, K. A. Pratt, P. B. Shepson, A. P. Ault, Inland sea spray aerosol transport and incomplete chloride depletion: Varying degrees of reactive processing observed during SOAS. *Environ. Sci. Technol.* **51**, 9533–9542 (2017).

55. J. C. F. Lo, A. K. H. Lau, J. C. H. Fung, F. Chen, Investigation of enhanced cross-city transport and trapping of air pollutants by coastal and urban land-sea breeze circulations. *J. Geophys. Res. Atmos.* **111**, 14104 (2006).

56. K. Müller, D. Hübner, S. Huppertsberg, T. P. Knepper, D. Zahn, Probing the chemical complexity of tires: Identification of potential tire-borne water contaminants with high-resolution mass spectrometry. *Sci. Total Environ.* **802**, 149799 (2022).

57. M. E. Balmer, H.-R. Buser, M. D. Müller, T. Poiger, Occurrence of some organic UV filters in wastewater, in surface waters, and in fish from Swiss lakes. *Environ. Sci. Technol.* **39**, 953–962 (2005).

58. M. J. Reid, K. H. Langford, J. Mørland, K. V. Thomas, Quantitative assessment of time dependent drug-use trends by the analysis of drugs and related metabolites in raw sewage. *Drug Alcohol Depend.* **119**, 179–186 (2011).

59. P. Horký, R. Grabic, K. Grabicová, B. W. Brooks, K. Douda, O. Slavík, P. Hubená, E. M. S. Santos, T. Randák, Methamphetamine pollution elicits addiction in wild fish. *J. Exp. Biol.* **224**, jeb242145 (2021).

60. B. A. Williams, J. E. M. Watson, H. L. Beyer, C. J. Klein, J. Montgomery, R. K. Runting, L. A. Roberson, B. S. Halpern, H. S. Grantham, C. D. Kuempel, M. Frazier, O. Venter, A. Wenger, Global rarity of intact coastal regions. *Conserv. Biol.* **36**, e13874 (2022).

61. M. Shoeib, J. Schuster, C. Rauert, K. Su, S. A. Smyth, T. Harner, Emission of poly and perfluoroalkyl substances, UV-filters and siloxanes to air from wastewater treatment plants. *Environ. Pollut.* **218**, 595–604 (2016).

62. P. R. Tumminello, R. C. James, S. Kruse, A. Kawasaki, A. Cooper, I. Guadalupe-Diaz, K. L. Zepeda, D. R. Crocker, K. J. Mayer, J. S. Sauer, C. Lee, K. A. Prather, J. H. Slade, Evolution of sea spray aerosol particle phase state across a phytoplankton bloom. *ACS Earth Space Chem* **5**, 2995–3007 (2021).

63. J. H. Slade, T. M. Vanreken, G. R. Mwaniki, S. Bertman, B. Stirm, P. B. Shepson, Aerosol production from the surface of the Great Lakes. *Geophys. Res. Lett.* **37**, L18807 (2010).

64. J. Best, Anthropogenic stresses on the world's big rivers. *Nat. Geosci.* **12**, 7–21 (2019).

65. L. P. Wright, L. Zhang, I. Cheng, J. Aherne, G. R. Wentworth, Impacts and effects indicators of atmospheric deposition of major pollutants to various ecosystems-A review. *Aerosol Air Qual. Res.* **18**, 1953–1992 (2018).

66. A. W. Cooper, M. M. Rogers, K. J. Wiggin, J. H. Slade, We need a "keeling curve" approach for contaminants of emerging concern. *Environ. Sci. Technol.* **57**, 10147–10150 (2023).

67. Quality and Wastewater | UN-Water. https://unwater.org/water-facts/quality-and-wastewater/.

68. S. Ramos, V. Homem, A. Alves, L. Santos, A review of organic UV-filters in wastewater treatment plants. [Preprint] (2016). https://doi.org/10.1016/j.envint.2015.10.004.

69. J. B. Lamb, J. A. J. M. Van De Water, D. G. Bourne, C. Altier, M. Y. Hein, E. A. Fiorenza, N. Abu, J. Jompa, C. D. Harvell, Seagrass ecosystems reduce exposure to bacterial pathogens of humans, fishes, and invertebrates. *Science* **355**, 731–733 (2017).

70. N. Mimura, Sea-level rise caused by climate change and its implications for society. *Proc. Jpn. Acad. Ser. B Phys. Biol. Sci.* **89**, 281–301 (2013).

71. M. Ghanbari, M. Arabi, S. C. Kao, J. Obeysekera, W. Sweet, Climate change and changes in compound coastal-riverine flooding hazard along the U.S. coasts. *Earths Future* **9**, e2021EF002055 (2021).

72. Y. Li, Z. Hu, X. Liu, Y. Dong, Y. Wang, S. Zhang, Z. Xu, Q. Yang, Characteristics of bioaerosol emissions from a municipal wastewater treatment plant: Health risk assessment and microbial composition. *Sci. Total Environ.* **934**, 173096 (2024).

73. Y. Wang, W. Wang, X. Yu, Z. Wang, Z. Zhou, Y. Han, L. Li, Global diversity of airborne pathogenic bacteria and fungi from wastewater treatment plants. *Water Res.* **258**, 121764 (2024).

74. C. Tuholske, B. S. Halpern, G. Blasco, J. C. Villasenor, M. Frazier, K. Caylor, Mapping global inputs and impacts from of human sewage in coastal ecosystems. *PLOS ONE* **16**, e0258898 (2021).

75. M. Small, "Imperial beach closed almost 900 days due to Tijuana river sewage issue," *ABC 10 News San Diego* (2024).

76. S. D. and A. C. (Sedac), Percentage of Total Population Living in Coastal Areas. *United Nations*, 170–175 (2008).

77. International Boundary and Water Commission, Water Data Portal. https://waterdata.ibwc.gov/AQWebportal.

78. B. J. Turpin, J. J. Huntzicker, S. V. Hering, Investigation of organic aerosol sampling artifacts in the los angeles basin. *Atmos. Environ.* **28**, 3061–3071 (1994).

79. CDIP, Coastal Data Information Program, *Scripps Institution of Oceanography* (2022). http://cdip.ucsd.edu/themes/data/download/?station=155&stream=p1&sub_stream=p1&public=1&start=202001&end=202005&file_type=pm&download_mode=browser.

80. C. J. Gaston, P. K. Quinn, T. S. Bates, J. B. Gilman, D. M. Bon, W. C. Kuster, K. A. Prather, The impact of shipping, agricultural, and urban emissions on single particle chemistry

observed aboard the R/V Atlantis during CalNex. *J. Geophys. Res. Atmos.* **118**, 5003–5017 (2013).

81. NERRS, NOAA National Estuarine Research Reserve System, *NERSS* (2022). http://nerrsdata.org.

82. CDIP, Coastal Data Information Program, *Scripps Institution of Oceanography* (2022). https://cdip.ucsd.edu/themes/cdip?pb=1&u2=s:073:st:1&d2=p9.

83. D. Pereira, Wind Rose, MATLAB Central File Exchange (2017). https://mathworks.com/matlabcentral/fileexchange/47248-wind-rose.

84. A. F. Corral, H. Dadashazar, C. Stahl, E. Lou Edwards, P. Zuidema, A. Sorooshian, Source apportionment of aerosol at a coastal site and relationships with precipitation chemistry: A case study over the Southeast United States. *Atmosphere* **11**, 1212 (2020).

85. A. P. Ault, M. J. Moore, H. Furutani, K. A. Prather, Impact of emissions from the Los Angeles Port region on San Diego air quality during regional transport events. *Environ. Sci. Technol.* **43**, 3500–3506 (2009).

86. A. Stohl, M. Hittenberger, G. Wotawa, Validation of the Lagrangian particle dispersion model FLEXPART against large-scale tracer experiment data. *Atmos. Environ.* **32**, 4245–4264 (1998).

87. S. Saha, S. Moorthi, X. Wu, J. Wang, S. Nadiga, P. Tripp, D. Behringer, Y.-T. Hou, H. Chuang, M. Iredell, M. Ek, J. Meng, R. Yang, M. P. Mendez, H. van den Dool, Q. Zhang, W. Wang, M. Chen, E. Becker, NCEP Climate Forecast System Version 2 (CFSv2) 6-hourly Products. Research Data Archive at the National Center for Atmospheric Research, Computational and Information Systems Laboratory [Preprint] (2011). https://doi.org/10.5065/D61C1TXF.

88. X. Chen, F. Zhang, K. Zhao, Diurnal variations of the land–sea breeze and its related precipitation over South China. *J. Atmos. Sci.* **73**, 4793–4815 (2016).

89. S. T. K. Miller, B. D. Keim, R. W. Talbot, H. Mao, Sea breeze: Structure, forecasting, and impacts. *Rev. Geophys.* **41**, 1011 (2003).

90. A. M. J. Van Eijk, J. T. Kusmierczyk-Michulec, M. J. Francius, G. Tedeschi, J. Piazzola, D. L. Merritt, J. D. Fontana, Sea-spray aerosol particles generated in the surf zone. *J. Geophys. Res. Atmos.* **116**, 19210 (2011).

91. T. Dittmar, B. Koch, N. Hertkorn, G. Kattner, A simple and efficient method for the solid-phase extraction of dissolved organic matter (SPE-DOM) from seawater. *Limnol Oceanogr. Methods* **6**, 230–235 (2008).

92. L. Cancelada, R. R. Torres, J. Garrafa Luna, P. C. Dorrestein, L. I. Aluwihare, K. A. Prather, D. Petras, Assessment of styrene-divinylbenzene polymer (PPL) solid-phase extraction and non-targeted tandem mass spectrometry for the analysis of xenobiotics in seawater. *Limnol Oceanogr. Methods* **20**, 89–101 (2022).

93. D. Petras, J. J. Minich, L. B. Cancelada, R. R. Torres, E. Kunselman, M. Wang, M. E. White, E. E. Allen, K. A. Prather, L. I. Aluwihare, P. C. Dorrestein, Non-targeted tandem mass spectrometry enables the visualization of organic matter chemotype shifts in coastal seawater. *Chemosphere* **271**, 129450 (2021).

94. D. Petras, I. Koester, R. Da Silva, B. M. Stephens, A. F. Haas, C. E. Nelson, L. W. Kelly, L. I. Aluwihare, P. C. Dorrestein, High-resolution liquid chromatography tandem mass spectrometry enables large scale molecular characterization of dissolved organic matter. [Preprint] (2017). https://doi.org/10.3389/fmars.2017.00405.

95. M. C. Chambers, B. MacLean, R. Burke, D. Amodei, D. L. Ruderman, S. Neumann, L. Gatto, B. Fischer, B. Pratt, J. Egertson, K. Hoff, D. Kessner, N. Tasman, N. Shulman, B. Frewen, T. A. Baker, M. Y. Brusniak, C. Paulse, D. Creasy, L. Flashner, K. Kani, C. Moulding, S. L. Seymour, L. M. Nuwaysir, B. Lefebvre, F. Kuhlmann, J. Roark, P. Rainer, S. Detlev, T. Hemenway, A. Huhmer, J. Langridge, B. Connolly, T. Chadick, K. Holly, J. Eckels, E. W. Deutsch, R. L. Moritz, J. E. Katz, D. B. Agus, M. MacCoss, D. L. Tabb, P. Mallick, A cross-platform toolkit for mass spectrometry and proteomics. *Nat. Biotechnol.* **30**, 918–920 (2012).

96. T. Pluskal, S. Castillo, A. Villar-Briones, M. Orešič, MZmine 2: Modular framework for processing, visualizing, and analyzing mass spectrometry-based molecular profile data. *BMC Bioinformatics* **11**, 395 (2010).

97. M. Wang, J. J. Carver, V. V. Phelan, L. M. Sanchez, N. Garg, Y. Peng, D. D. Nguyen, J. Watrous, C. A. Kapono, T. Luzzatto-Knaan, C. Porto, A. Bouslimani, A. V. Melnik, M. J. Meehan, W. T. Liu, M. Crüsemann, P. D. Boudreau, E. Esquenazi, M. Sandoval-Calderón, R. D. Kersten, L. A. Pace, R. A. Quinn, K. R. Duncan, C. C. Hsu, D. J. Floros, R. G. Gavilan, K. Kleigrewe, T. Northen, R. J. Dutton, D. Parrot, E. E. Carlson, B. Aigle, C. F. Michelsen, L. Jelsbak, C. Sohlenkamp, P. Pevzner, A. Edlund, J. McLean, J. Piel, B. T. Murphy, L. Gerwick, C. C. Liaw, Y. L. Yang, H. U. Humpf, M. Maansson, R. A. Keyzers, A. C. Sims, A. R. Johnson, A. M. Sidebottom, B. E. Sedio, A. Klitgaard, C. B. Larson, C. A. P. Boya, D. Torres-Mendoza, D. J. Gonzalez, D. B. Silva, L. M. Marques, D. P. Demarque, E. Pociute, E. C. O'Neill, E. Briand, E. J. N. Helfrich, E. A. Granatosky, E. Glukhov, F. Ryffel, H. Houson, H. Mohimani, J. J. Kharbush, Y. Zeng, J. A. Vorholt, K. L. Kurita, P. Charusanti, K. L. McPhail, K. F. Nielsen, L. Vuong, M. Elfeki, M. F. Traxler, N. Engene, N. Koyama, O. B. Vining, R. Baric, R. R. Silva, S. J. Mascuch, S. Tomasi, S. Jenkins, V. Macherla, T. Hoffman, V. Agarwal, P. G. Williams, J. Dai, R. Neupane, J. Gurr, A. M. C. Rodríguez, A. Lamsa, C. Zhang, K. Dorrestein, B. M. Duggan, J. Almaliti, P. M. Allard, P. Phapale, L. F. Nothias, T. Alexandrov, M. Litaudon, J. L. Wolfender, J. E. Kyle, T. O. Metz, T. Peryea, D. T. Nguyen, D. VanLeer, P. Shinn, A. Jadhav, R. Müller, K. M. Waters, W. Shi, X. Liu, L. Zhang, R. Knight, P. R. Jensen, B. Palsson, K. Pogliano, R. G. Linington, M. Gutiérrez, N. P. Lopes, W. H. Gerwick, B. S. Moore, P. C. Dorrestein, N. Bandeira, Sharing and community curation of mass spectrometry data with Global Natural Products Social Molecular Networking. *Nat. Biotechnol.* **34**, 828–837 (2016).

98. C. Hansch, A. Leo, D. Hoekman, Hydrophobic, electronic, and steric constants. American Chemical Society [Preprint] (1995).

99. National Academies of Sciences, Engineering, and Medicine, *Review of Fate, Exposure, and Effects of Sunscreens in Aquatic Environments and Implications for Sunscreen Usage and Human Health* (The National Academies Press, Washington, DC, 2022); https://doi.org/10.17226/26381.

100. C. A. Downs, E. Kramarsky-Winter, R. Segal, J. Fauth, S. Knutson, O. Bronstein, F. R. Ciner, R. Jeger, Y. Lichtenfeld, C. M. Woodley, P. Pennington, K. Cadenas, A. Kushmaro, Y. Loya, Toxicopathological effects of the sunscreen UV filter, oxybenzone (benzophenone-3), on coral planulae and cultured primary cells and its environmental contamination in Hawaii and the U.S. Virgin Islands. *Arch. Environ. Contam. Toxicol.* **70**, 265–288 (2016).

101. S. Li, J. Wen, B. He, J. Wang, X. Hu, J. Liu, Occurrence of caffeine in the freshwater environment: Implications for ecopharmacovigilance. *Environ. Pollut.* **263**, 114371 (2020).

102. L. R. Vieira, A. M. V. M. Soares, R. Freitas, Caffeine as a contaminant of concern: A review on concentrations and impacts in marine coastal systems. *Chemosphere* **286**, 131675 (2022).

103. IBWC, Binational Water Quality Study of the Tijuana River and Adjacent Canyons and Drains: December 2018 to November 2019. (2020).

104. J. Brahney, N. Mahowald, M. Prank, G. Cornwell, Z. Klimont, H. Matsui, K. A. Prather, Constraining the atmospheric limb of the plastic cycle. *Proc. Natl. Acad. Sci. U.S.A.* **118**, e2020719118 (2021).

105. L. Dsikowitzky, I. Nordhaus, C. H. Sujatha, P. S. Akhil, K. Soman, J. Schwarzbauer, A combined chemical and biological assessment of industrial contamination in an estuarine system in Kerala, India. *Sci. Total Environ.* **485-486**, 348–362 (2014).

106. O. L. Mayol-Bracero, O. Rosario, C. E. Corrigan, R. Morales, I. Torres, V. Pérez, Chemical characterization of submicron organic aerosols in the tropical trade winds of the caribbean using gas chromatography/mass spectrometry. *Atmos. Environ.* **35**, 1735–1745 (2001).

107. O. E. Johnson, H. Patel, G. M. Miskelly, J. D. Rindelaub, Drug substances in the air of a New Zealand city. *Atmos. Pollut. Res.* **14**, 101750 (2023).

108. P. C. Raynor, A. Bartekova, J. G. Griggs, M. F. Simcik, J. L. Adgate, Airborne diazinon concentrations during and after outdoor spray application. *J. Occup. Environ. Hyg.* **7**, 506–515 (2010).

109. M. Viana, C. Postigo, X. Querol, A. Alastuey, M. J. López De Alda, D. Barceló, B. Artíñano, P. López-Mahia, D. García Gacio, N. Cots, Cocaine and other illicit drugs in airborne particulates in 104urban environments: A reflection of social conduct and population size. *Environ. Pollut.* **159**, 1241–1247 (2011).

110. R. Lewis, E. Schwartz, *Sea Salt Aerosol Production: Mechanisms, Methods, Measurements and Models—A Critical Review* (American Geophysical Union, 2004; http://agu.org/books/gm/v152/, vol. 152 of *Geophysical Monograph Series*).

111. C. L. Archer, M. Z. Jacobson, Evaluation of global wind power. *J. Geophys. Res. Atmos.* **110**, D12110 (2005).

112. U.S. EPA. Chapter 6 - Inhalation Rates, Exposure Factors Handbook 2011 Edition (Final Report). U.S. Environmental Protection Agency, Washington, DC, EPA/600/R-09/052F, 2011.
